# Jobseekers’ skills and job search behaviour

**DOI:** 10.1186/s41937-025-00142-9

**Published:** 2025-10-20

**Authors:** Conny Wunsch, Felix Rochlitz, Patrick Arni

**Affiliations:** 1 Faculty of Business and Economics, University of Basel, Peter Merian Weg 6, PO Box CH-4002, Basel, Switzerland; 2 CESifo, Munich, Germany; 3 DIW, Berlin, Germany; 4 IZA, Bonn, Germany; 5 Faculty of Business and Economics, University of Basel, Basel, Switzerland; 6 ZHAW School of Management and Law, Winterthur, Switzerland; 7 University of Bristol, Bristol, UK; 8 CAFE Aarhus University, Aarhus, Denmark; 9 HEC Lausanne, Lausanne, Switzerland

**Keywords:** Skills, Skill gaps, Job search

## Abstract

This paper uses novel linked survey and administrative data for jobseekers in Switzerland to study jobseekers’ skills, potential skill gaps, and their job search behaviour. Based on a realized sample of survey participants that is better educated and has better employment prospect than the overall population of jobseekers, we find that women and older jobseekers are most at risk of lacking digital skills, while low education and little work experience are risk factors associated with lacking professional and interdisciplinary skills. We further document that the willingness of jobseekers to deviate from their last job in terms of skill requirements is relatively low and that they are reluctant to accept wage losses. However, jobseekers with potential skill gaps do tailor their search strategy to their skill profile. Our results provide interesting insights for policy makers and practitioners in the public employment service who seek to support jobseekers in navigating modern labour markets with rapidly changing skill requirements.

## Introduction

The digital transformation is rapidly changing the skills required in the labour market. Digital skills are becoming more important for labour market success across all occupations (e.g. Autor et al., 2003; Zobrist & Brandes, 2017; Deming, 2017; Deming & Kahn, 2018). To prevent skill mismatch in the labour market, labour supply needs to keep up with changing skill requirements. Unemployed workers are particularly vulnerable in the digital transformation. Workers in jobs with decreasing demand and workers who lack demanded skills face a higher risk of becoming unemployed, and they are more likely to have difficulties finding a new job. Moreover, given the rapid change in skill requirements, human capital depreciation during unemployment is a major concern.

The objective of this paper is to study jobseekers’ skills, potential skill gaps, and their job search behaviour. For this purpose, we collected novel survey data of jobseekers in Switzerland that we link to their administrative data as well as occupation-level data on skill requirements, wages, labour demand and jobseekers. We contacted the full population of jobseekers who were registered with selected regional offices of the Swiss public employment service in the German-speaking area between September 2022 and January 2023. Compared to the target population, the participants in our online survey are better educated and have better digital skills and employment prospects. It is important to keep this in mind when interpreting our finding. Our analysis proceeds in five steps. Firstly, we document heterogeneity in jobseekers’ perceptions about their digital, professional and interdisciplinary skills. Secondly, we use jobseekers skill self-assessments to construct a novel measure of potential skill gaps. For digital skills, this measure equals the difference between the jobseeker’s self-assessment and a prediction based on education and the characteristics of the last job, including the required level of digital skills. For professional and interdisciplinary skills, we measure coverage of the 14 most important skills in the jobseeker’s last occupation and the 14 most relevant alternative occupations. Thirdly, we investigate which types of jobseekers are most likely to have potential skill gaps. Fourthly, we study jobseekers’ job search strategies and how they relate to potential skill gaps. In the last step, we analyse how potential skill gaps, job search strategies and job search outcomes are related.

We document several interesting findings. Firstly, we observe substantial variation in how individuals assess their own skills, even after accounting for differences in education and occupational sorting. Consistent with previous research on gender disparities in digital competencies (see Van Laar et al., 2017, for a review), our findings show that women rate their digital skills significantly lower than men. They also report lower self-assessments of professional skills but higher ratings for interdisciplinary skills. We also find a negative relationship between age and self-assessed skills across most domains—particularly digital skills—with interdisciplinary skills being the only exception. These results support concerns that older jobseekers may be less equipped for the demands of the digital economy. Secondly, our measures of potential skill gaps indicate that women and older individuals are particularly vulnerable to digital skill deficits, while low educational attainment and limited work experience are key risk factors for lacking professional and interdisciplinary skills. Thirdly, our analysis shows that jobseekers tend to favour positions similar to their previous roles and are generally reluctant to accept lower wages—especially men. In contrast, older jobseekers appear more willing to compromise on salary. The likelihood of pursuing a job decreases as the skill profile diverges from a jobseeker’s prior experience, even when the overlap is still substantial. Fourthly, despite a general reluctance to change occupations, our measure of potential skill gaps predicts jobseekers' willingness to adjust their job search to occupations with skill requirements that better fit their skill profile. Finally, conditional on search strategies, we find that individuals with higher-than-predicted digital skills exit the public employment service (PES) system more quickly. Conversely, negative digital skill gaps and lower professional skill coverage do not significantly correlate with deregistration rates. However, lower coverage in interdisciplinary skills is linked to a higher likelihood of remaining registered with the PES 12 months after the initial sampling.

Our paper adds to a growing literature that documents digital skills on the labour supply side (see Van Laar et al., 2017, for a review). Studies that aim to identify skill mismatch on the individual level are still rare, though. They are mostly limited to self-assessments by workers of whether their skills or formal qualifications fit those required by their current job. This results in very broad and typically one-dimensional measures of mismatch without linking them to specific skills. Moreover, they relate to the specific job held, which ignores alternative jobs workers could take. Studies of skill shortages from the perspective of employers typically use firm aggregates (McGuinness et al., 2018). Few cases study employees’ perceptions of underskilling (e.g. McGuinness and Ortiz 2016), and some studies propose methods for measuring skill mismatch on the level of occupations (e.g. Sahin et al., 2014; Modestino et al., 2023). Recently, Bächli et al. (2024) proposed a method to measure skill mismatch of jobseekers in 12 selected general skills that are relevant in all occupations, although to a different degree. Similar to what we do, this measure is based on the comparison of skill requirements in occupations and an online assessment of jobseekers’ skills. Our approach uses skill self-assessments of jobseekers but covers a much broader range of job-relevant skills. Our paper complements (Amosa, 2023) who document jobseekers digital and social skills and potential skill mismatch in Eastern Switzerland and the cantons of Aargau, Zug and Zurich based on a survey with 797 participants. Besides larger regional coverage of our sample, we go considerably beyond their work by studying how perceived skills and potential skill gaps relate to job search behaviour and job search outcomes.

By studying skill perceptions, our paper is related to the literature on the role of beliefs about the returns to search and job search outcomes. A growing literature shows that beliefs about relevant aspects of job search affect job search behaviour and job search outcomes (e.g. Laguna, 2013; Spinnewijn, 2015; Arni, 2019; Spinnewijn, 2015; Mueller et al., 2021). We extend this literature by analysing the role of beliefs about own job-relevant skills. With this, our paper is related to the literature that documents biases in beliefs about skills (e.g. Svenson 1981; Camerer and Lovallo, 1999; DellaVigna, 2009). We further add to the literature that is interested in the relationship between job search strategies and job search outcomes more generally,^1^ and to the literature that studies how the breadth of search affects job search outcomes in particular.^2^ By investigating how jobseekers’ beliefs about their skills and potential skill deficits relate to job search behaviour, our paper contributes to a better understanding of why jobseekers pursue different job search strategies.

The remainder of this paper is structured as follows: Section 2 describes our data and study population. Section 3 documents how jobseekers’ perceive their skills and how this varies with their characteristics. Here, we also introduce our measure of potential skill gaps and document heterogeneity therein. Section 4 describes jobseekers’ job search behaviour, and how this relates to potential skill gaps. Section 5 studies the relationship between potential skill gaps, search strategies, and job search outcomes. The final section concludes. An appendix provides additional material and estimation results.

## Data

### Individual-level data

**Survey data.** We conducted an online survey of jobseekers registered with the public employment service in selected regional employment offices (REOs) in the German-speaking area of Switzerland.^3^ The goal of this survey was to study job search behaviour and job search outcomes of registered jobseekers in Switzerland in the light of the digital transformation. The target population of the survey consists of the full stock of jobseekers registered with the relevant REOs at the end of, respectively, September 2022, November 2022 and January 2023, with an email address in the administrative data. Jobseekers received an email invitation from us to participate in the survey in November 2022, January 2023 and February 2023, respectively. One reminder was sent about one to two weeks after the initial invitation. The overall response rate to the survey was 8.4%, and the median completion time in our analysis sample was 21 min. In the analysis, we only include jobseekers that were aged 15–59 and had been registered for less than 18 months at the sampling date. We exclude formerly self-employed jobseekers as well as students, interns and apprentices.

**Administrative data.** Subject to jobseekers’ consent, we link the survey data to administrative data from the PES at the time of sampling that includes the demographic characteristics of the jobseekers, their registration date, information on the job looked for (employment level, occupation) and detailed information on their last job. Additionally, we have the information whether our survey participants were registered with the PES six and twelve months after initial sampling.

**Job search behaviour.** To measure job search behaviour, we presented jobseekers who consent to data linkage with a personalized list of 15 occupations that may fit their profile and ask them how frequently they search in these occupations.^4^ Answering this question was mandatory, i.e. participants could not proceed with the survey unless they answered. We also allow them to enter other occupations they look for in a text field.^5^ The list of 15 occupations includes the jobseeker’s last occupation and up to three occupations looked for according to the administrative data. We add occupations based on two criteria. First, we use the most frequent combinations of previously held and found occupations observed among the full population of jobseekers for the years 2017–2022 in the administrative data. Second, we include up to five occupations with the largest overlap with the last job in terms of required skills. Specifically, we measure the share of basic and specific professional skills as well as interdisciplinary skills that the jobseeker’s last occupation has in common with each other ISCO 4-digit occupation according to skill data provided by the Austrian labour Market Service (AMS, see Sect. 2.2 for details). We also calculate overlap in terms of required digital skills as the last occupations’s skill level divided by the level of the respective other occupation. Then, we compute an overall measure of overlap by adding the respective shares, giving basic professional skills a weight of two, specific professional skills a weight of three and interdisciplinary and digital skills a weight of one each. We then normalize the sum by the maximum possible value of 7 to obtain the final overlap measure. We exclude occupations where the overlap measure is less than 0.25. We rank all occupations in descending order of the observed combination frequency and skill overlap and pick the first 11–14 to obtain a list of 15 occupations for each jobseeker. In the following, we refer to these occupations as the top 15.

**Jobs search intensity and outcomes.** Within the survey, we collect data on the number of hours typically spent on job search activities per week and the number of applications sent out in the past four weeks. As job search outcomes, we ask for the number of interview invitations received in the past four weeks. Additionally, we observe job search duration at sampling and at the time of participation in the survey. From the administrative data, we further know whether or not a person is registered as jobseeker with the PES six and twelve months after initial sampling.

### Occupation-level data

We describe job search strategies using the characteristics of all top 15 occupations and jobseekers’ last job. Specifically, we characterize the occupations in terms of skill requirements, average wages, vacancies and number of jobseekers.

**Skill requirements.** The Austrian Labour Market Service (AMS) publishes detailed information on required skills for each occupation in the Austrian occupation classification together with a mapping into the ISCO-08 classification on the 4-digit level.^6^ On the one hand, the AMS reports lists of required basic professional skills, specific professional skills and interdisciplinary skills. The purpose of these lists is to comprehensively describe the competencies required in the occupation. Skills that are required in multiple occupations are labelled in exactly the same way. We exploit this feature to construct measures of skill overlap across occupations. The skill lists are based on occupation-specific training regulations and curricula, text analysis of job ads as well as expert panels that evaluate the skills extracted from the other sources. The information is reviewed regularly and updated when necessary. On the other hand, the AMS publishes the level of digital skills required in a given occupation. It is measured on a scale from 1 (basic skills) over 2 (independent application) and 3 (advanced) to 4 (highly specialized). It is evaluated in six different dimensions that are based on the competence model by Narosy et al. (2018). This model is adapted to Austria from the competence framework of the European Union outlined in Carretero et al. (2017), see BMDW (2021) for details. It comprises the following dimensions: (1) essentials; (2) information and data literacy; (3) communicating and collaborating; (4) creating digital content; (5) security; (6) problem solving and continuous learning. The evaluation of the six dimensions is used to obtain an overall level of required digital skills for each occupation. In addition to the sources used for the skill lists, the evaluation of digital skill requirements is based on an extensive review of existing literature, national and international projects about digital skills required in different occupations, as well as various industry-specific expert workshops with firm representatives. The AMS uses regular text analysis of job ads and expert panels to monitor whether skill levels need to be adjusted.^7^ The data on skill requirements we use here is from October 2022.

**Average wages.** We use data from the Swiss Labour Force survey to obtain average wages for each ISCO 4-digit occupation. We pool the data for the years 2021–2023 to obtain a sufficiently large sample of 115’766 employed workers. We construct full-time-equivalent annual wages based on the reported annual gross wage and hours worked per week. We use wages to account for the financial attractiveness of different occupations.

**Number of vacancies.** We use the number of vacancies per occupation to characterize labour demand and employment prospects in different occupations. We measure the number of vacancies in the sampling month of each jobseeker using data from the job search platform of the PES (job-room.ch). The platform contains all vacancies reported to the PES by firms. Jobseekers registered with the PES are encouraged to use this platform for their job search. The platform also offers various e-services that jobseekers can use for interacting with the PES. For example, they can submit their monthly prove of compliance with job search requirements for unemployment insurance claims. In our sample, 88% of jobseekers are registered on the platform.

**Number of jobseekers.** As a measure of competition from other jobseekers, we measure the total number of jobseekers per occupation at the time of sampling from the full population in the REOs that were targeted with our survey.

**Table 1:** Characteristics of the study sample

	(1)	(2)	(3)	(4)	(5)	(6)
	Survey		Representative sample		Diff.	
	Mean	Std.	Mean	Std.	(3)-(1)	P-value
	Jobseeker characteristics					
Woman	0.482	0.500	0.466	0.499	$-$0.017	0.128
Age (years)	42.93	10.59	38.99	10.92	$-$3.941	0.000
Not Swiss	0.448	0.497	0.544	0.498	0.095	0.000
Compulsory schooling	0.202	0.401	0.324	0.468	0.122	0.000
Basic vocational education	0.371	0.483	0.425	0.494	0.054	0.000
Advanced vocational education	0.159	0.365	0.108	0.311	$-$0.050	0.000
University or equivalent	0.269	0.444	0.143	0.350	$-$0.126	0.000
	Characteristics of last job					
High share non-routine cognitive	0.499	0.500	0.357	0.479	$-$0.142	0.000
High share non-routine manual	0.465	0.499	0.643	0.479	0.178	0.000
High share routine cognitive	0.486	0.500	0.474	0.499	$-$0.013	0.255
High share routine manual	0.258	0.437	0.312	0.463	0.054	0.000
Management function	0.126	0.332	0.055	0.229	$-$0.070	0.000
Expert function	0.618	0.486	0.584	0.493	$-$0.034	0.002
Support function	0.256	0.437	0.361	0.480	0.105	0.000
Low-tech manufacturing	0.087	0.282	0.095	0.293	0.008	0.204
High-tech manufacturing	0.064	0.245	0.056	0.230	$-$0.008	0.138
Less knowledge-intensive services	0.401	0.490	0.431	0.495	0.030	0.006
Knowledge-intensive services	0.389	0.488	0.304	0.460	$-$0.086	0.000
Construction	0.058	0.235	0.114	0.317	0.055	0.000
	Work experience					
Less than 1 year	0.106	0.308	0.121	0.326	0.015	0.040
1–3 years	0.170	0.376	0.228	0.420	0.058	0.000
More than 3 years	0.723	0.447	0.651	0.477	$-$0.073	0.000
	Search-related characteristics					
Job search duration (days)	98	117	125	131	26.95	0.000
Registered as unemployed	0.538	0.499	0.566	0.496	0.028	0.011
Observations	2736		8,050			

All variables are from the administrative records and measured at the sampling date. The representative sample is a 10% random sample from the target population of the survey at the respective sampling date. Except for age and job search duration, all variables are shares. We classify occupations according to their task content. Mihaylov and Tijdens (2019) construct an index of the task intensity of each 4-digit ISCO-08 occupation in four dimensions: routine and non-routine manual and cognitive tasks. We flag occupations as high intensity for a given index if they are above the median of the index. As occupations may be above the median in more than one index, they can show up in more than one high-intensity group. We classify industries according to Beerli et al. (2021)

### Study sample

Our analysis sample consists of 2,736 jobseekers who participated in our survey between November 2022 and February 2023 and agreed to link their survey data to their administrative records. Table 1 summarizes the characteristics of this sample and compares it to a representative 10% random sample of the target population of the survey. Our sample consists of 48% women and the average age is 43 years, which is 4 years older than the representative sample. The latter is reflected in a higher share of jobseekers with more than three years of work experience. As the survey was only offered in German, foreigners are under-represented by almost 10 percentage points. As a direct consequence, our sample contains a higher share of jobseekers with a university or equivalent degree, fewer jobseekers with a high share of routine manual tasks or only a support function in the last job, and a higher share from knowledge-intensive service sectors. Furthermore, by construction, we only observe jobseekers who have access to a digital device and sufficient digital skills to participate in an online survey. Thus, our sample consists of jobseekers with better digital skills and employment prospects compared to the target population. This is reflected in an average job search duration at sampling of 98 days compared to 125 days in the representative sample, and a lower share of jobseekers registered as unemployed. In our analysis, we abstain from reweighing our sample to the representative sample for transparency.

## Jobseekers’ self-assessment of their skills

### Measurement and descriptive statistics

In the survey, jobseekers self-assessed their digital, professional and interdisciplinary skills on a 7-point scale from very bad to very good allowing for the answer “don’t know”. The questions are mandatory, i.e. participants were unable to proceed with the survey unless they answer. Figure 4 in Appendix A shows the original question in the survey tool for digital skills (in German).^8^

**Fig. 1:**
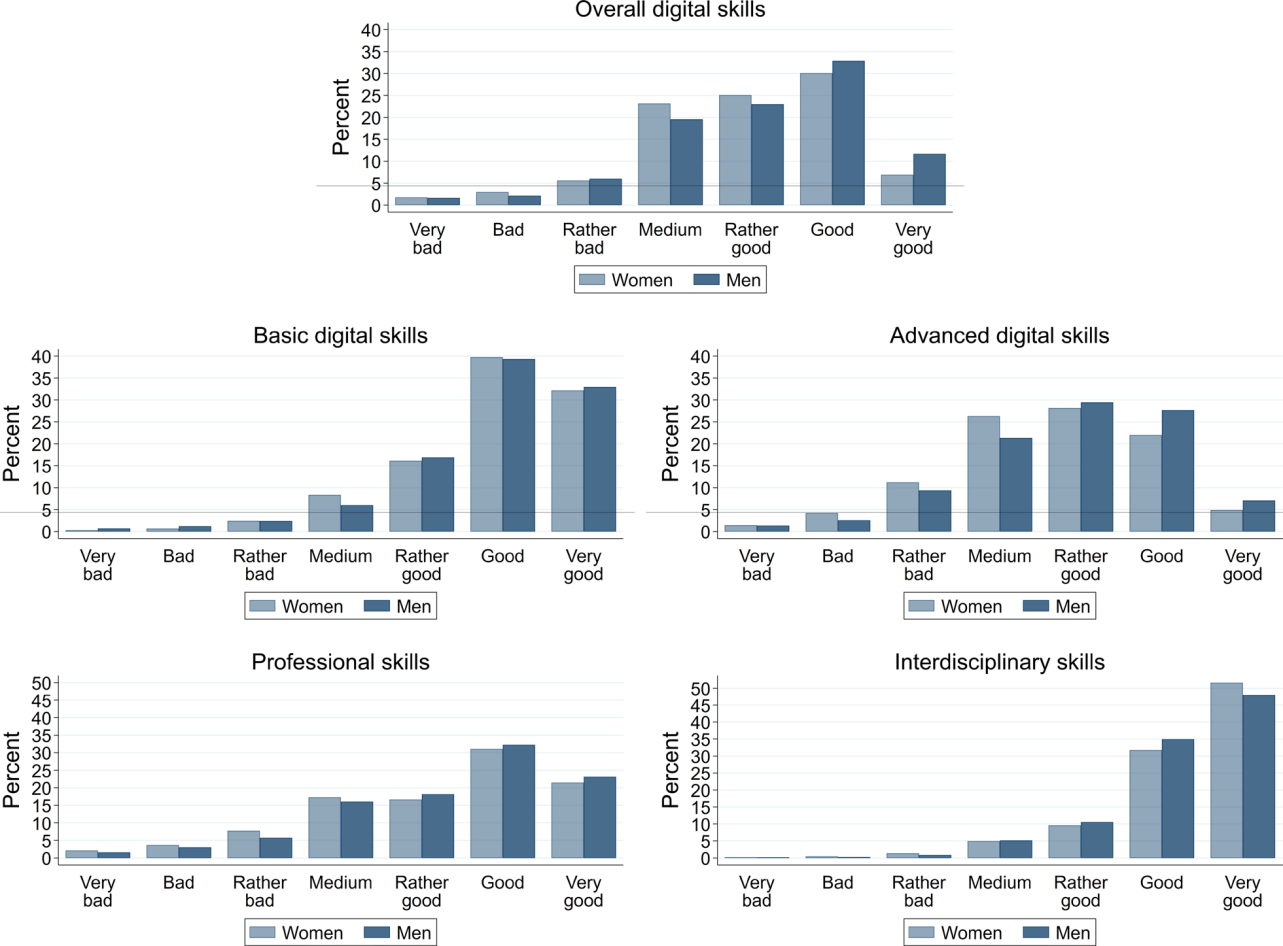
Distribution of self-assessed skills. *Note*: The figure shows histograms for overall, basic and advanced digital skills as well as for professional and interdisciplinary skills. The latter two pool the 14 items per jobseeker

**Digital skills.** Jobseekers self-assess their digital skills overall as well as ten specific skill items that are part of the competence model of Narosy et al. (2018) and can be aggregated to the six skill dimensions of the model. We use this framework to ensure comparability to the data on occupation-specific skill requirements. In Table 2, we report the sample averages of each skill item in the survey for the full sample as well as separately for men and women. In column (4) we display the gender difference and in column (5) the p-value for a test of statistical significance of this difference. We further indicate the dimension of the competence model of Narosy et al. (2018) to which the respective item belongs in italics. We also group them into basic and advanced digital skills for future reference. Figure 1 shows the distribution of self-reported skills for digital skills overall, basic and advanced digital skills, which we obtain by averaging the individual items listed in Table 2. Table 12 in Appendix B shows the pairwise correlations between all digital skill items and Fig. 5 in Appendix C the distribution for each individual item.

We find that the large majority of jobseekers rate their basic digital skills as good or very good. This is not surprising, given that the jobseekers in our sample needed to have sufficient digital skills to participate in our online survey. Most gender differences are small and not statistically significant on conventional levels. The only larger difference occurs for *(4) Making online purchases or sales*, with women rating themselves better on average than men (p-value .058). For advanced digital skills, the distribution is shifted much more to lower levels, and women rate their skills consistently lower than men. With the exception of *(7) Protecting devices and personal data*, all of these differences are statistically significant at p-values of .000 to .079. The differences are particularly large for *(8) Solving technical problems* and *(9) Solving tasks or problems using digital technologies*. Both skills are highly correlated. The distribution of the overall rating of digital skills (11) in Fig. 1 shows few jobseekers with a rating below medium and significantly better evaluations for men than for women. The correlation of the overall rating with the individual items is high, ranging from .57 to .80.

**Table 2:** Self-assessment of skills

		(1)	(2)	(3)	(4)	(5)
					Diff.	
		All	Men	Women	(3)-(2)	P-value
Basic digital skills						
*Essentials:*						
(1)	Using digital devices (e.g. computers)	5.89	5.92	5.86	-.064	.173
*Information and data literacy:*						
(2)	Searching, interpreting & handling data & digital content	5.72	5.75	5.69	-.067	.161
*Communicating and collaborating:*						
(3)	Communicating, collaborating & sharing information using digital technologies	5.80	5.80	5.80	-.006	.901
(4)	Making online purchases or sales	5.77	5.72	5.82	.096	.058
Advanced digital skills						
*Creating digital content:*						
(5)	Developing & programming digital content	3.65	3.72	3.57	-.155	.023
(6)	Automating processes	4.70	4.75	4.64	-.110	.077
*Security:*						
(7)	Protecting devices and personal data	5.12	5.13	5.10	-.025	.666
*Problem solving and continuous learning:*						
(8)	Solving technical problems	4.67	5.01	4.29	-.726	.000
(9)	Solving tasks or problems using digital technologies	4.95	5.11	4.77	-.347	.000
(10)	Recognizing own gaps in digital skills	5.10	5.15	5.05	-.098	.079
(11)	Digital competencies overall	5.03	5.12	4.93	-.188	.000
Observations		2,736	1,416	1,320		
(12)	Professional skills	5.25	5.32	5.18	$-$0.136	0.000
(13)	Interdisciplinary skills	6.21	6.19	6.23	0.035	0.001
Observations (14 items per jobseeker)		38,304	19,824	18,480		

The table shows sample averages of the respective survey item. Each item can have one of the following values: 1=very bad, 2=bad, 3=rather bad, 4=medium, 5=rather good, 6=good, 7=very good. For professional and interdisciplinary skills, there are 14 items pooled per jobseeker. Column (4) shows the difference in means between women and men. Column (5) reports the p-value for the test of statistical significance of this difference

**Professional and interdisciplinary skills.** For professional and interdisciplinary skills, we present jobseekers with a personalized list of 14 skills each that are most important in the top 15 relevant occupations based on the data on skills required in each occupation from the Austrian Labour Market Service.^9^ Again, jobseekers can choose between seven levels from very bad to very good. The lower part of Table 2 reports the sample averages of skill self-assessments over all 14 items per jobseeker and the lower part of Fig. 1 their distribution. Most jobseekers evaluate their professional skills from medium to very good, with about a third of skill items rated as good and about 20% as very good. The ratings of interdisciplinary skills are very high. Almost half of the items are assessed as very good by jobseekers and another third as good. Interestingly, ratings of professional skills are significantly lower for women than for men, while women evaluate their interdisciplinary skills significantly higher than men.

### Heterogeneity

To describe heterogeneity in skill ratings, we run regressions of jobseekers’ skill self-assessment on demographics (gender, age, nationality), education, and characteristics of jobseekers’ last job (required level of digital skills, function, sector). We further include work experience as a proxy for potential skill accumulation. To capture potential skill depreciation, we add search duration at the survey date as well as a dummy variable for whether or not the jobseeker is registered as unemployed at the sampling date. As dependent variables, we use the skill evaluations for overall digital skills, basic and advanced digital skills, as well as professional and interdisciplinary skills. For the latter two, we pool all items such that we have 14 observations per jobseeker. We report the results in Table 3.

**Table 3:** Heterogeneity in self-assessed skills

	(1)	(2)	(3)	(4)	(5)
	Overall	Basic	Advanced	Profes-	Interdis-
	digital	digital	digital	sional	ciplinary
	skills	skills	skills	skills	skills
Digital skills required in the last job	0.373***	0.389***	0.279***	0.043	0.074***
	(0.050)	(0.041)	(0.049)	(0.034)	(0.024)
Management function	0.072	0.145***	0.046	0.199***	0.193***
	(0.069)	(0.049)	(0.071)	(0.046)	(0.026)
Support function	$-$0.169**	$-$0.117*	$-$0.077	$-$0.105**	$-$0.070**
	(0.078)	(0.063)	(0.073)	(0.051)	(0.033)
Low-tech manufacturing	0.026	0.097	0.062	$-$0.047	$-$0.025
	(0.094)	(0.071)	(0.088)	(0.060)	(0.039)
High-tech manufacturing	0.380***	0.273***	0.275***	0.041	0.049
	(0.090)	(0.067)	(0.098)	(0.070)	(0.043)
Knowledge-intensive services	0.017	0.029	$-$0.001	$-$0.009	0.028
	(0.058)	(0.045)	(0.056)	(0.037)	(0.024)
Construction	$-$0.455***	$-$0.535***	$-$0.402***	$-$0.137*	$-$0.180***
	(0.125)	(0.113)	(0.118)	(0.075)	(0.056)
Compulsory schooling	$-$0.190**	$-$0.261***	$-$0.100	$-$0.087	$-$0.050
	(0.085)	(0.071)	(0.079)	(0.055)	(0.035)
Advanced vocational education	0.222***	0.250***	0.089	0.109**	0.029
	(0.069)	(0.054)	(0.068)	(0.048)	(0.031)
University or equivalent	0.272***	0.249***	0.078	0.082*	0.047*
	(0.064)	(0.047)	(0.064)	(0.043)	(0.028)
Work experience less than 1 year	$-$0.012	0.044	0.028	$-$0.141**	$-$0.046
	(0.095)	(0.075)	(0.089)	(0.062)	(0.042)
Work experience 1–3 years	$-$0.003	0.023	0.036	$-$0.077	$-$0.050
	(0.075)	(0.059)	(0.071)	(0.049)	(0.032)
Job search duration at survey date	$-$0.001	$-$0.005	0.003	$-$0.004	$-$0.006**
	(0.006)	(0.004)	(0.005)	(0.004)	(0.003)
Registered as unemployed	0.061	0.026	0.028	$-$0.025	$-$0.062***
	(0.050)	(0.039)	(0.048)	(0.032)	(0.021)
Female	$-$0.188***	$-$0.034	$-$0.268***	$-$0.109***	0.060***
	(0.052)	(0.042)	(0.051)	(0.034)	(0.022)
Age	$-$0.016***	$-$0.022***	$-$0.018***	$-$0.003*	0.001
	(0.003)	(0.002)	(0.002)	(0.002)	(0.001)
Not Swiss	0.110**	$-$0.006	0.192***	0.144***	0.059**
	(0.054)	(0.042)	(0.052)	(0.035)	(0.023)
Constant	4.890***	5.852***	4.864***	5.345***	6.040***
	(0.186)	(0.150)	(0.176)	(0.120)	(0.080)
Observations	2,532	2,617	2,587	34,463	36,168
Adjusted R-squared	0.111	0.183	0.062	0.014	0.023

Reference categories are: expert function, less knowledge-intensive services, basic vocational education and more than 3 years of work experience. Robust standard errors in parentheses. *** *p*<0.01, ** *p*<0.05, * *p*<0.1. For professional and interdisciplinary skills, there are 14 skill items per person and standard errors are clustered at the individual level

**Characteristics of last job.** Skill requirements vary strongly across occupations as we show in Fig. 6 in Appendix D. Therefore, we expect that the characteristics of the last job are important drivers of heterogeneity, especially for digital skills. As expected, we find that the level of digital skills required in the last job is strongly and positively associated with self-assessments of digital skills. We also find a positive correlation with professional and interdisciplinary skill ratings, which is in line with skill self-assessments being positively correlated. Other job characteristics have explanatory power as well. Workers with a management function rate their skills higher than those with an expert function, significantly so in terms of basic digital, professional and interdisciplinary skills. Workers with a support function report considerably lower skills, with statistically significant differences in all skill dimensions except advanced digital skills. Compared to the reference group of less knowledge-intensive service sectors, digital skill ratings are higher for jobseekers from high-tech manufacturing and lower for all skills for construction workers.

**Education.** We expect that skills are positively correlated with education, which is confirmed by our results. Jobseekers with a university or equivalent degree or with advanced vocational training report consistently better skills than those with basic vocational education. Conversely, jobseekers with only compulsory schooling rate their skills considerably lower. The coefficients are statistically significant for overall and basic digital skills. For professional, they are significant for university graduates and advanced vocational education, for interdisciplinary skills only for university graduates.

**Work experience.** Workers tend to acquire more skills the longer they stay on the same job. Therefore, jobseekers with the same last job and the same level of education may differ in their skills depending on their work experience. The administrative data only contain a very crude measure of overall work experience. It only differentiates three categories: less than one year, 1–3 years and more than 3 years, where the majority of jobseekers falls into the last group (72%, Table 1). Moreover, it does not distinguish between experience in different occupations. We find no significant association of this crude measure with digital skill ratings, but lower skill evaluations of jobseekers with less than one year of work experience for professional skills.

**Search duration and status.** Skills may depreciate the longer a worker remains jobless. This may be particularly relevant for digital skills given how fast digital technologies change. Moreover, jobseekers become negatively selected with longer unsuccessful search. In line with expectations, we find lower skill ratings with longer job search duration and when being registered as unemployed for interdisciplinary skills. However, our results show no significant correlation with digital and professional skill ratings. There are several possible explanations. First, the digital skill items in the survey do not refer to specific digital technologies that may have emerged or become more important. Second, jobseekers may not be aware that their skills depreciate. Third, only 54% of jobseekers registered with the PES are unemployed (Table 1). Finally and probably most importantly, average search duration is low in our sample with 98 days.

**Demographics.** The variables discussed so far resemble objective factors that are expected to drive actual skill differences. Significant coefficients for the remaining demographic variables indicate that there are differences in skill self-assessments that are not explained by these other variables. In particular, they are neither explained by sorting into different jobs, nor by differences in ability to the extent to which they are captured by different levels of education. Demographics will matter if different groups invest differentially in skills conditional on education and occupational sorting, or if their subjective skill ratings differ systematically from their actual skills.

**Gender.** Even after accounting for occupational sorting, our results show lower skill ratings for women than for men for overall and advanced digital skills. This is in line with previous evidence on a gender divide in digital skills and skill investments that has been found in many different contexts (see Van Laar et al., 2017). It is also in accordance with research that finds that men over-estimate their digital skills more than women (e.g. Palczynska and Rynko, 2021). Interestingly, we find a negative and significant gender gap for professional skills but a positive one for interdisciplinary skills. This is in line with evidence that women tend to under-estimate their IQ whereas men tend to over-estimate it, while the reverse is observed for emotional intelligence (Reilly et al., 2022; Furnham & Robinson, 2023).

**Age.** We document a negative association of skill ratings with age for all skill dimensions except interdisciplinary skills. The estimated negative coefficients are particularly large for digital skills. This is in line with previous findings (e.g. Bhattacharjee et al., 2020) and corroborates concerns that older jobseekers may under-invest in skills, especially in digital skills, and may be less well prepared for the digital transformation.

**Nationality.** We find interesting differences by nationality. With the exception of basic digital skills, jobseekers without the Swiss nationality evaluate their skills as significantly better than Swiss jobseekers. It is important to keep in mind, though, that foreigners in our sample are positively selected compared to all foreign jobseekers, because they have sufficient German language skills to participate in our survey.

**Robustness.** Table 16 in Appendix F shows that our results are robust to adding more explanatory variables. They remain unchanged when adding ISCO occupations on the 1-digit level and task content of occupations to account for occupational sorting in more detail. They are also robust to including an indicator for being married, dummy variables for the greater regions of Switzerland, and month of sampling to capture time effects.

### Potential skill mismatch

In the remainder of the paper, we aim to study how differences in perceived skills are associated with differences in job search strategies and outcomes. Ideally, we would like to investigate whether actual and perceived skill mismatch with respect to the last job is associated with job search in occupations that better match jobseekers’ actual or perceived skills. Measurement of *actual* skill mismatch requires objective information on the skills of jobseekers that are important in relevant jobs. Measuring *perceived* skill mismatch requires information on jobseekers’ *beliefs* about own skills and the skills required in potential jobs. Our data include actual skill requirements in the last job and potential alternatives. We also observe subjective skill self-assessments that may be subject to biased beliefs about own skills. However, we lack information on jobseekers’ actual skills and their beliefs about skill requirements of jobs. Moreover, for digital skills, self-assessments are measured on a different scale than the job requirements. For professional and interdisciplinary skills, we only observe evaluations for a subset of all potentially relevant skills. In the following, we propose measures of potential skill gaps based on the information we observe.

**Potential gaps in digital skills.** To obtain a proxy for skill requirements in the last job on the same scale as the skill self-assessments, we use a prediction based on objective descriptors. We use all jobseekers with more than 3 years of work experience and regress their skill self-assessment on education and the job characteristics in Table 3 plus dummy variables for the ISCO 1-digit occupations. Most importantly, the predictors include the level of digital skills required in the last job. We use the estimated coefficients to predict, for all jobseekers in the sample, the skill level that would be expected given their last job and educational attainment. The difference between jobseekers’ skill self-assessment and the prediction reflects *potential* skill gaps. Differences can occur if jobseekers’ actual skills deviate from the predicted level, if jobseekers have biased beliefs about their skills, or if our prediction misses important factors.

**Fig. 2:**
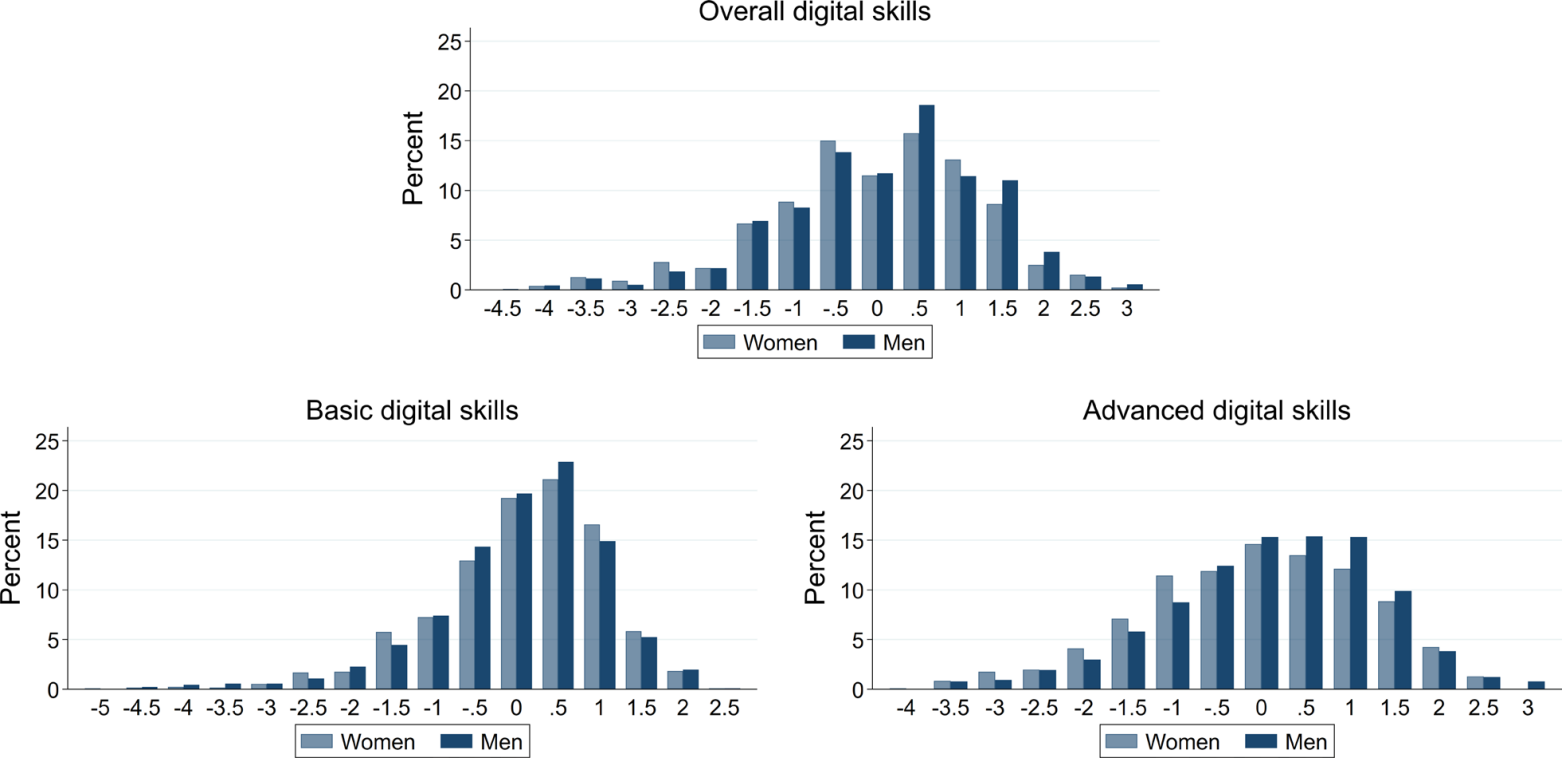
Potential gaps in digital skills. *Note*: The figure shows the percentage share of jobseekers for binned values of potential gaps for overall, basic and advanced digital skills

Figure 2 displays the distribution of the potential gaps for overall, basic and advanced digital skills separately by gender. The distributions are skewed towards positive gaps, especially for basic digital skills, where most jobseekers rate their skills as good or very good. This pattern is in line with evidence on over-confidence regarding own ability (see Pulford and Colman, 1997; Furnham and Rawles, 1999; Ames and Kammrath, 2004; Bhandari and Deaves, 2006; Moore and Healy, 2008). Gender differences are difficult to assess visually for overall and basic digital skills. For advanced digital skills, we see considerably fewer positive gaps and more negative gaps for women than men.

**Fig. 3:**
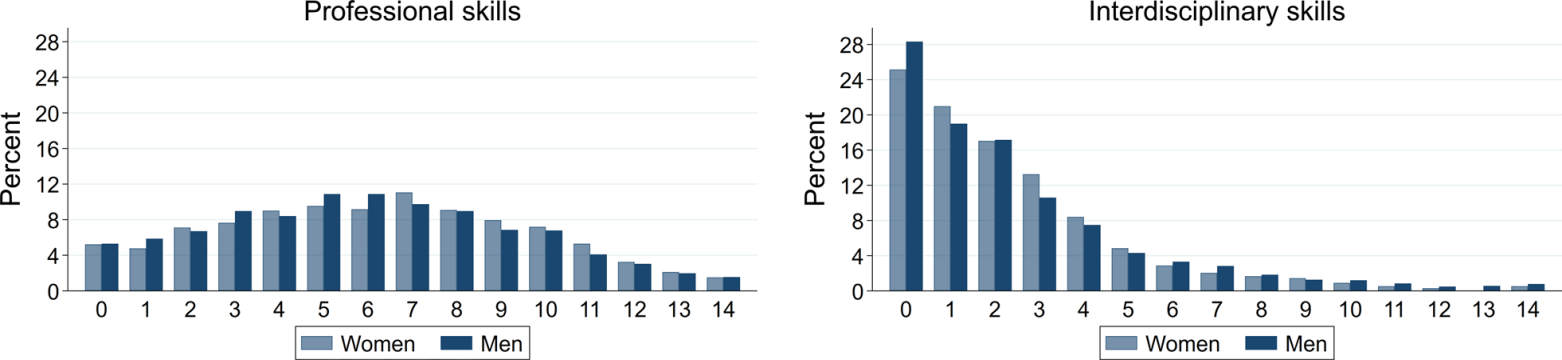
Potential gaps in professional and interdisciplinary skills. *Note*: The figure shows the percentage share of jobseekers who rated the indicated number of skills as less than good

**Fig. 4:**
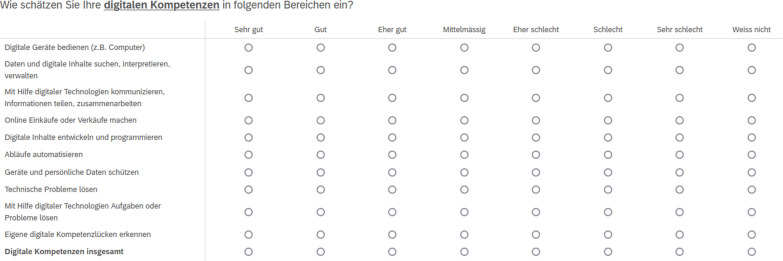
Survey question on digital skills

**Fig. 5:**
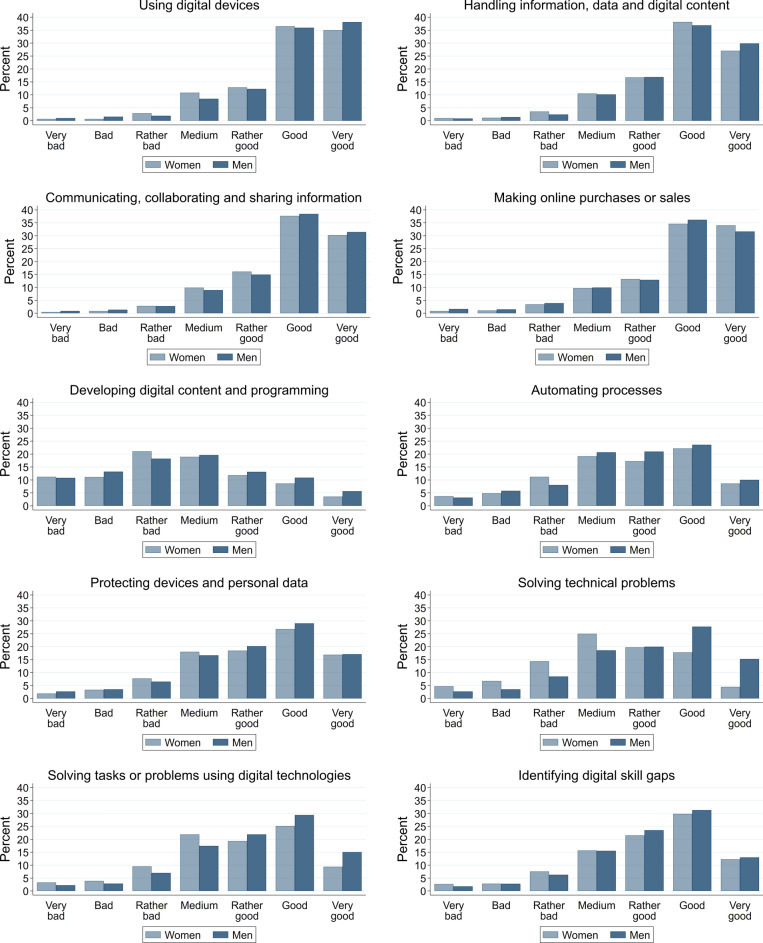
Distribution of self-assessed skills for single items. *Note*: The figure shows the percentage share of jobseekers for each stated level of digital skills

**Fig. 6:**
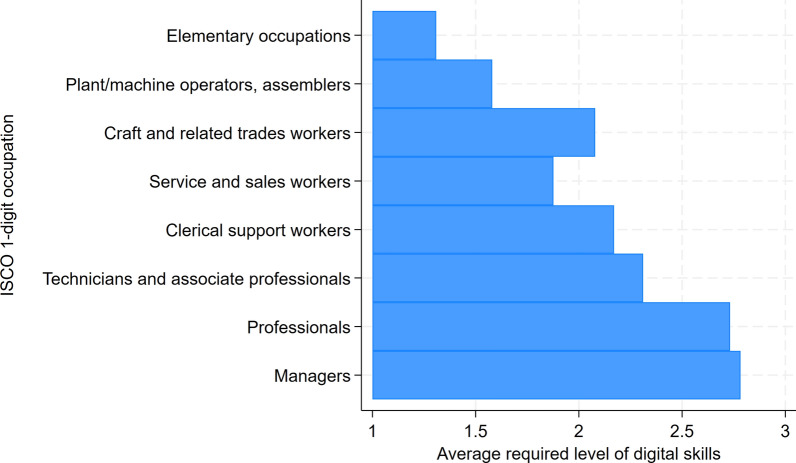
Average level of required digital skills by ISCO 1-digit occupation. *Note*: The figure shows the average required level of digital skills by ISCO 1-digit occupation of the last job of the jobseekers in our sample

**Coverage of relevant professional and interdisciplinary skills.** As a proxy for potential mismatch regarding professional and interdisciplinary skills, we count the number of skills (out of 14), where jobseekers rate themselves as less than good. The 14 professional and interdisciplinary skills jobseekers assess comprise those that are most important in their last job and relevant alternatives (i.e. in the top 15 occupations). The count measure is based on the idea that jobseekers are more likely to lack skills that are important in relevant occupations and, therefore, may be constrained in their job opportunities the fewer of these skills they cover. Figure 3 shows the distribution of the measure for professional and interdisciplinary skills, respectively. There is substantial variation in coverage of professional skills. For interdisciplinary skills, coverage is very high with the majority of jobseekers not covering at most two of the 14 most important skills.

**Potential skill gaps and intentions to strengthen skills.** At the end of the survey, we included a question whether jobseekers intend to strengthen their digital, professional or interdisciplinary skills. In Appendix F Table 15, we show the results of regressions of an indicator variable for the answer yes to this question on potential skill gaps. Specifically, we include one indicator variable each for the difference between the digital skill self-assessment and the prediction being lower than the lower tercile ($-$0.395) or higher than the upper tercile (0.665), respectively. We also add the count measures for the number of professional and interdisciplinary skills in which jobseekers rate themselves less than good. We run this regression both with and without other control variables. The results show that potential skill deficits are either uncorrelated with these intentions or negatively correlated. This suggests that jobseekers may not be aware of these gaps or that low skill self-assessments coincide with a low willingness to invest in the respective skills.

**Heterogeneity.** Policy makers are particularly concerned about jobseekers who lack relevant skills. To investigate which types of jobseekers are more likely to have potential skill deficits, we study in Table 4 how negative potential gaps in digital skills and lack of coverage of professional and interdisciplinary skills correlate with jobseeker characteristics. In columns (1)–(3), we regress an indicator for negative deviations from the predicted level of digital skills on gender, age, nationality, job search experience at the survey date, registration status, education and work experience. In columns (4)–(5), we use the count measure for lacking, respectively, professional and interdisciplinary skills as dependent variable. To account for the correlation of potential skill gaps, columns (1)–(3) additionally control for lacking professional and interdisciplinary skills, while columns (4)–(5) control for potential gaps in overall digital skills.

In line with our results for the level of skill ratings, we find that more women have negative gaps in all digital skill dimensions than men. This speaks to female jobseekers having lower skills than men, or that women are more likely to under-estimate their skills. The share of jobseekers with negative gaps increases with age in all digital skill dimensions. This finding is in line with skill depreciation or lower skill investments at older ages. Potential lack of professional and interdisciplinary skills is negatively correlated with gender and age, significantly so in most cases. Foreign jobseekers are less likely to lack skills in all dimensions, significantly so for advanced digital skills and professional skills. Longer job search duration and being registered as unemployed is associated with significantly lower coverage of interdisciplinary skills. In line with expectations, lower coverage of both professional and interdisciplinary skills is significantly associated with lower education and less work experience. Jobseekers with the lowest level of education are also more likely to lack advanced digital skills. Taken together, our results suggest that women and older jobseekers are most at risk of lacking digital skills, while low education and little work experience are risk factors associated with lacking professional and interdisciplinary skills.

**Table 4:** Heterogeneity in potential skill deficits

	(1)	(2)	(3)	(4)	(5)
	Overall	Basic	Advanced	Profes-	Interdis-
	digital	digital	digital	sional	ciplinary
	skills	skills	skills	skills	skills
Female	0.082***	0.031*	0.081***	$-$0.038	$-$0.360***
	(0.019)	(0.018)	(0.018)	(0.128)	(0.100)
Age	0.006***	0.009***	0.006***	$-$0.016**	$-$0.018***
	(0.001)	(0.001)	(0.001)	(0.007)	(0.005)
Not Swiss	$-$0.009	$-$0.002	$-$0.047**	$-$0.517***	$-$0.021
	(0.020)	(0.019)	(0.019)	(0.138)	(0.106)
Job search duration at survey date	$-$0.002	$-$0.000	$-$0.002	0.014	0.022*
	(0.002)	(0.002)	(0.002)	(0.015)	(0.012)
Registered as unemployed	$-$0.032*	0.007	$-$0.025	0.247*	0.358***
	(0.019)	(0.018)	(0.018)	(0.127)	(0.098)
Compulsory schooling	0.027	0.005	0.058**	$-$0.233	0.236
	(0.027)	(0.026)	(0.026)	(0.197)	(0.162)
Advanced vocational education	0.003	0.033	0.024	$-$0.429**	$-$0.319**
	(0.027)	(0.026)	(0.026)	(0.184)	(0.139)
University or equivalent	0.003	0.042*	0.026	$-$0.429***	$-$0.600***
	(0.023)	(0.023)	(0.023)	(0.155)	(0.116)
Less than 1 year	$-$0.043	$-$0.084***	$-$0.060**	0.721***	0.453**
	(0.031)	(0.029)	(0.030)	(0.234)	(0.186)
1–3 years	$-$0.003	$-$0.046*	$-$0.019	0.277	0.328**
	(0.026)	(0.025)	(0.025)	(0.181)	(0.154)
Constant	$-$0.117**	$-$0.283***	$-$0.171***	6.999***	3.112***
	(0.050)	(0.048)	(0.048)	(0.340)	(0.275)
Observations	2,532	2,617	2,587	2,532	2,532
Adjusted R-squared	0.158	0.173	0.191	0.165	0.142

Reference categories are: basic vocational education and more than 3 years of work experience. Robust standard errors in parentheses *** * p*<0.01, ** * p*<0.05, * * p*<0.1. In columns (1)–(3), the dependent variable is an indicator for negative deviations from the predicted level of digital skills. In columns (4)–(5), the dependent variable is the count measure for lacking professional or interdisciplinary skills. In columns (1)–(3), we additionally control for lacking professional and interdisciplinary skills, while columns (4)–(5) control for potential gaps in overall digital skills

## Job search strategies

### Occupational differences and job search decisions

When jobseekers decide in which occupations to look for jobs, many different aspects may matter. Our survey design allows us to study to which extent differences in the characteristics of occupations relative to jobseekers’ last occupation drive job search decisions. We do not observe all potentially relevant occupations and differences across occupations. However, for the top 15 occupations, we can assess wages and the determinants of labour market tightness as key economic drivers that are central in job search models, as well as differences in skill requirements.

We compile a dataset that contains one observation for each top 15 occupation per jobseeker. For each occupation, we measure the difference to the last job in terms of average wages, number of vacancies, number of jobseekers and required level of digital skills. We also add a measure of overlap in required professional skills and interdisciplinary skills. It equals the share of the skills required in the respective occupation that are also required in the last occupation of the jobseeker. Table 5 provides descriptive statistics for this dataset. It reports the mean in column (1), the standard deviation (2), the 25th (3), 50th (4) and 75th (5) percentiles, as well as the minimum (6) and maximum (7).

**Table 5:** Descriptive statistics for top 15 occupations

	(1)	(2)	(3)	(4)	(5)	(6)	(7)
	Mean	Std	.25	.50	.75	Min	Max
Difference in digital skills	0.012	0.253	$-$0.133	0.000	0.111	$-$0.667	2.000
Difference in basic digital skills	0.008	0.234	$-$0.132	0.000	0.113	$-$0.661	1.949
Difference in advanced digital skills	0.003	0.236	$-$0.133	0.000	0.100	$-$1.000	1.949
Overlap in professional skills	0.816	0.210	0.718	0.885	0.974	0.000	1.000
Overlap in interdisciplinary skills	0.784	0.219	0.667	0.833	1.000	0.000	1.000
Wage difference	0.021	0.271	$-$0.136	0.000	0.128	$-$0.717	2.144
Vacancy difference	12.796	98.487	$-$0.824	$-$0.143	1.874	$-$1.000	3292
Jobseeker difference	4.830	36.941	$-$0.792	0.000	1.280	$-$1.000	3047
Observations	41,205						
Individuals	2,747						

The sample consists of all occupations in the list of 15 provided to jobseekers. All differences are measured in percent/100 of the level of the last job. Overlap measures the share that the last job has in common with the respective occupation

The differences to the last job are small on average and zero at the median for the digital skill requirements, wages, and number of jobseekers. But there is considerable variation. At the 25th percentile, digital skill requirements are 13% lower than for the last job, while they are 10–11% larger at the 75th percentile. Wage differences range from -14% to 13% at the 25th and 75th percentile, respectively. The differences in the number of vacancies and jobseekers can be quite large. They reach around -80% at the 25th percentile and far more than 100% at the 75th percentile. The overlap with the last job in terms of professional and interdisciplinary skills is high with, respectively, 82% and 78% on average and 89% and 83% at the median. Overlap is already high at the 25th percentile with 72% and 67%, respectively, and it reaches 97% and 100% at the 75th percentile. This supports our claim that the top 15 list of occupations comprises jobs that are relevant alternatives for the jobseeker.

**Gender differences.** Due to occupational sorting, men and women differ considerably with respect to their last occupation, which implies that the top 15 occupations differ across gender. To assess these difference, Tables 13 and 14 in Appendix E provide the same statistics separately for men and women, respectively. Gender differences are mostly small at the mean and the median. However, the 25th percentiles are systematically lower for men than for women, i.e. negative deviations are larger. The 75th percentiles are larger for women than for men for the digital skill requirements and wage differences, meaning that positive deviations are larger. But the upper quartiles are very similar for skill overlap, and considerably larger for men for the determinants of labour market tightness.

**Job search decisions.** To assess whether occupational differences matter for job search decisions, we regress an indicator for whether or not the jobseeker searches in a given top 15 occupation on these differences. We include individual fixed effects to take out all individual heterogeneity, such that we only exploit within-person variation in the differences to the jobseeker’s last job. We separately include the absolute value of positive and negative deviations from the last job to allow for differential associations, with no difference to the last job as reference (note that the last job is part of the top 15 list). Using absolute values implies that the estimated coefficients measure how the probability of searching in a given occupation changes when the deviations from the last job become more positive or negative, respectively. To align reference groups, we use gaps in skill overlap defined as one minus overlap for professional and interdisciplinary skills. Instead of the actual value of gaps in interdisciplinary skills, we use the residuals from a regression of the gaps on gaps in professional skills because both variables are highly correlated with a value of .756 (p-value .000). Table 6 displays the results for the full sample as well as separately for men and women. For the interpretation of the results, it is important to keep in mind that lack of overlap in skills can mean both lower and higher skill requirements than the last job. Hence, differences in how demanding jobs are will mainly be captured by wage differences.

**Table 6:** Probability to search for a top 15 occupation

	(1)	(2)	(3)
	All	Men	Women
Positive wage difference ^+^	$-$0.08586***	$-$0.02430	$-$0.15047***
	(0.01806)	(0.02734)	(0.02419)
Negative wage difference^+^	$-$0.43867***	$-$0.55953***	$-$0.23109***
	(0.02932)	(0.03681)	(0.04777)
Positive vacancy difference	$-$0.00000	$-$0.00001	0.00002
	(0.00004)	(0.00005)	(0.00006)
Negative vacancy difference	$-$0.08543***	$-$0.07756***	$-$0.09929***
	(0.00784)	(0.01074)	(0.01156)
Positive jobseeker difference	0.00023	0.00026	0.00012
	(0.00015)	(0.00019)	(0.00022)
Negative jobseeker difference	$-$0.21900***	$-$0.21792***	$-$0.21181***
	(0.00857)	(0.01251)	(0.01177)
Positive difference in digital skills^+^	$-$0.02332	$-$0.09230***	0.07222**
	(0.01882)	(0.02391)	(0.02925)
Negative difference in digital skills^+^	$-$0.22599***	$-$0.15403***	$-$0.32955***
	(0.02378)	(0.03261)	(0.03414)
Gap in professional skills^+^	$-$0.15035***	$-$0.11651***	$-$0.19754***
	(0.01560)	(0.02098)	(0.02308)
Gap in interdisciplinary skills^+^	$-$0.21733***	$-$0.29420***	$-$0.11287***
	(0.02270)	(0.02942)	(0.03623)
Constant	0.64395***	0.64943***	0.64088***
	(0.00479)	(0.00641)	(0.00710)
Individual fixed effects	Yes	Yes	Yes
Observations	39,635	20,536	19,099
Individuals	2,696	1,400	1,296
Adjusted R-squared	0.18292	0.20501	0.16315

The sample consists of all occupations in the list of 15 provided to jobseekers. The dependent variable is one if the person searches in the occupation and zero otherwise. The explanatory variables measure the difference to the last occupation held by the jobseeker. The reference group is no difference. Negative differences enter positively such that the estimated coefficient measures the change in the search probability when the difference becomes more negative. Gaps in interdisciplinary skills are measured as the residuals from a regression of the actual value on gaps in professional skills. Robust standard errors in parentheses. *** *p*<0.01, ** *p*<0.05, * *p*<0.1. ^+^ indicates that the difference in coefficients across gender is statistically significant on the 5% level

**Results.** We find that jobseekers prefer jobs that are more similar to their last job. Larger negative deviations from the last job in any of the dimensions significantly and substantially reduce the probability of searching in an occupation. Jobseekers seem to be particularly adverse to wage losses. Interestingly, men seem considerably more reluctant to accept wage losses than women. Moreover, wage gains, which are associated with more demanding jobs, seem to discourage women from searching in these jobs but not men. Positive deviations from the last job in terms of labour demand and competition from other jobseekers are not significantly associated with search probabilities.^10^ Jobseekers seem to be reluctant to downgrade in terms of digital skill requirements, interestingly women more so than men. Moreover, higher digital skill requirements than the last job correlate with a higher search probability for women but a lower one for men. Smaller overlap in terms of professional and interdisciplinary skills significantly reduces the search probability. Thus, jobseekers are more reluctant to pursue jobs the more their skill profiles differ from their last job, even among jobs with relatively large skill overlap. Interestingly, men are more discouraged by differences in interdisciplinary skills than women, while the reverse holds for professional skills (p-value .098). Taken together, our results suggest that the willingness to change occupation is relatively low among jobseekers and that reluctance to accept wage losses and differences in skill requirements seem to be the most relevant factors.

### Characteristics of searched occupations

In the next step of the analysis, we use the characteristics of the occupations in the top 15 list in which jobseekers report looking for work to define different job search strategies. Here, we aggregate across all searched occupations per jobseeker using the search frequencies as weights. We measure these frequencies on a 6-point scale with the values never (0), less than once per month (1), at least once per month (2), once per week (3), several times per week (4), and every day (5). We use these values divided by the sum over all 15 occupations as normalized weights to construct weighted averages over the characteristics of the jobs looked for. Table 7 provides descriptive statistics for these characteristics. We report means for the full sample and by gender, as well as gender differences and p-values for their statistical significance.

**Table 7:** Descriptive statistics on searched occupations

	(1)	(2)	(3)	(4)	(5)
	All	Men	Women	Diff. (3)-(2)	P-value
Difference in overall digital skills	$-$0.008	$-$0.032	0.018	0.049	0.000
Difference in basic digital skills	$-$0.010	$-$0.035	0.017	0.052	0.000
Difference in advanced digital skills	$-$0.010	$-$0.030	0.012	0.042	0.000
Overlap in professional skills	0.837	0.836	0.838	0.003	0.524
Overlap in interdisciplinary skills	0.815	0.813	0.818	0.005	0.344
Wage difference	0.026	0.009	0.044	0.035	0.000
Vacancy difference (median)	0.235	0.545	0.040	$-$0.505	0.000
Jobseeker difference (median)	0.312	0.613	0.009	$-$0.602	0.000
Observations	2,634	1,371	1,263		

Differences are in levels for the digital skill requirements. Wage, vacancy and jobseeker differences are measured in percent/100 of the level of the last job. Overlap measures the share that the last job has in common with the respective occupation. If not indicated otherwise, columns (1)–(3) contain means

We find that on average, jobseekers search in occupations that have slightly lower digital skill requirements than the last job but pay 2.6% higher wages. They have both more vacancies and jobseekers, suggesting that the searched occupations are larger. Average overlap in terms of professional and interdisciplinary skills is high with 84% and 82%, respectively, and it does not vary by gender. Interestingly, only men but not women downgrade with respect to digital skill requirements on average. The positive wage differences are very small for men, but relatively large with 4.4% on average for women. In contrast, the positive vacancy (and jobseeker) differences are much larger for men than for women. The gender differences we observe are in line with strong gender-specific occupational sorting.

To further characterize job search strategies, we define different measures that indicate how broadly jobseekers search and how strongly they are willing to deviate from their last job in terms of skill requirements and wages. Table 8 lists these measures and presents descriptive statistics in the same way as Table 7. On average, jobseekers search in 46.5% or 7 out of the 15 jobs from the top 15 list, with men covering a somewhat higher share than women. This relatively high share further supports the relevance of the occupations included in the top 15 list. About one-third of jobseekers each search in occupations that require more than 5% lower or higher digital skills on average. Interestingly, fewer women downgrade in terms of digital skill requirements than men and more upgrade. 30% of jobseekers aspire occupations that on average overlap less than 80% in terms of required professional skills, while 32.9% search quite narrowly with more than 90% overlap. Jobseekers deviate more in terms of interdisciplinary skills, with 36% with less than 80% overlap and 25.3% with more than 90%. Only 16.7% of jobseekers pursue jobs that on average pay more than 10% less than the last occupation, and 23.8% aspire jobs that pay more than 10% more. Interestingly and in contrast to the results in Table 6, fewer women than men pursue jobs with possible wage losses while more aspire higher paying jobs. It is important to keep in mind, though, that Table 6 is a within-person analysis, while Table 8 shows raw cross-sectional differences. Moreover, Table 6 studies the extensive margin of job search decisions, while Table 8 looks at differences conditional on searching in a given occupation, with more frequently searched occupations receiving a higher weight.

**Table 8:** Descriptive statistics on search strategies

	(1)	(2)	(3)	(4)	(5)
	All	Men	Women	Diff. (3)-(2)	P-value
Share of top 15 searched	0.465	0.474	0.455	$-$0.019	0.035
More than 5% lower digital skills	0.335	0.355	0.314	$-$0.042	0.024
More than 5% higher digital skills	0.332	0.303	0.365	0.062	0.001
Overlap in professional skills <80%	0.304	0.310	0.298	$-$0.012	0.492
Overlap in professional skills >90%	0.329	0.319	0.340	0.021	0.253
Overlap in interdisciplinary skills <80%	0.360	0.371	0.348	$-$0.023	0.221
Overlap in interdisciplinary skills >90%	0.253	0.265	0.239	$-$0.026	0.130
Wage more than 10% lower	0.167	0.194	0.138	$-$0.055	0.000
Wage more than 10% higher	0.238	0.216	0.263	0.047	0.005
Observations	2,736	1,416	1,320		

Columns (1)–(3) show the mean of the respective variable. Except for "Share of top 15 searched", all variables characterize the search frequency weighted average of searched occupations compared to the last occupation held by the jobseeker

**Correlations with other occupation characteristics.** For the interpretation of our results, it is important to understand to which extent search strategies correlate with other characteristics of the searched occupations. Table 17 in Appendix F shows the results of a regression of each strategy measure on the difference to the last job in terms of wages, vacancies, jobseekers, and skill requirements, leaving out the characteristic that defines the respective strategy. We find that searching in more of the top 15 occupations is associated with smaller differences in the number of vacancies, jobseekers and the required level of digital skills as well as larger differences in professional and interdisciplinary skill requirements. Downgrading in terms of digital skill requirements is associated with lower wages, more competition from other jobseekers, higher overlap in terms of professional skills but lower overlap in interdisciplinary skills. We find exactly the opposite for higher aspirations in terms of digital skills. Thus, jobseekers who pursue more (less) digitally demanding jobs seek jobs with better (worse) pay and employment prospects. A more narrow search in terms of both professional and interdisciplinary skill requirements is associated with smaller differences in the other skill requirements and better employment prospects (fewer jobseekers, more vacancies), while we observe the opposite for a broader search. Interestingly, both broader and more narrow search in terms of professional skill requirements are uncorrelated with wage differences, while both a broad and a narrow search in terms of interdisciplinary skill requirements is associated with wage losses. The willingness to accept wage losses is associated with more competition from other jobseekers, lower digital skill requirements and larger overlap in terms of professional skills, but smaller overlap in interdisciplinary skills. In contrast, aspiring higher wages correlates with higher digital skill requirements but none of the other occupation characteristics.

### Heterogeneity in job search strategies

We now study which types of jobseekers pursue which search strategy. Here, we are particularly interested in the question whether jobseekers with potential skill gaps seek jobs that potentially better fit their skill profile than their last job. Unfortunately, we cannot measure whether the jobseeker’s last job was a good match or not. We do not observe jobseekers’ actual skills such that we cannot compare them to the requirements of the last job. We also do not know whether they have been laid off or quit their job voluntarily.

To this end, we regress each strategy measure on one indicator variable each for the difference between the digital skill self-assessment and the prediction being lower than the lower tercile ($-$0.395) or higher than the upper tercile (0.665), respectively. Moreover, we include the count measures for the number of professional and interdisciplinary skills in which jobseekers rate themselves less than good. To characterize heterogeneity further, we add other characteristics of the jobseeker (gender, age, nationality, search duration and status, education, work experience). Table 9 displays the results. The regressions additionally control for the characteristics of the last job (required level of digital skills, function, sector, ISCO 1-digit occupation, task content of occupations) and month of sampling to account for heterogeneity driven by occupational sorting and time effects.

**Table 9:** Heterogeneity in job search strategies

	Share of	Digital skills		Professional skills		Interdisciplinary skills		Wage	
	top 15	More than	More than	Overlap	Overlap	Overlap	Overlap	More than	More than
	searched	5% lower	5% higher	<80%	>90%	<80%	>90%	10% lower	10% higher
	(1)	(2)	(3)	(4)	(5)	(6)	(7)	(8)	(9)
Higher-than-predicted digital skills	0.013	$-$0.032*	0.014	0.005	0.002	$-$0.002	$-$0.002	$-$0.019	0.058***
	(0.011)	(0.019)	(0.018)	(0.022)	(0.022)	(0.023)	(0.021)	(0.016)	(0.020)
Lower than predicted digital skills	$-$0.003	0.037*	$-$0.061***	$-$0.016	0.018	$-$0.029	0.021	0.015	$-$0.029
	(0.011)	(0.020)	(0.018)	(0.022)	(0.022)	(0.023)	(0.021)	(0.018)	(0.019)
Uncovered professional skills	$-$0.007***	$-$0.001	$-$0.001	0.006*	$-$0.003	0.005	0.002	0.006***	0.001
	(0.002)	(0.003)	(0.003)	(0.003)	(0.003)	(0.003)	(0.003)	(0.002)	(0.003)
Uncovered interdisciplinary skills	0.004	0.009***	$-$0.003	$-$0.002	$-$0.005	$-$0.000	$-$0.003	0.000	$-$0.002
	(0.002)	(0.003)	(0.003)	(0.004)	(0.004)	(0.004)	(0.004)	(0.003)	(0.003)
Female	$-$0.027***	0.035**	0.000	0.016	$-$0.013	0.020	$-$0.041**	$-$0.006	$-$0.001
	(0.010)	(0.017)	(0.016)	(0.019)	(0.020)	(0.020)	(0.018)	(0.015)	(0.018)
Age	$-$0.000	$-$0.000	$-$0.001	0.000	$-$0.001	$-$0.001	$-$0.000	0.001*	$-$0.003***
	(0.000)	(0.001)	(0.001)	(0.001)	(0.001)	(0.001)	(0.001)	(0.001)	(0.001)
Not Swiss	0.002	0.039**	$-$0.026	0.009	$-$0.004	$-$0.009	$-$0.007	0.025	$-$0.023
	(0.010)	(0.018)	(0.016)	(0.020)	(0.021)	(0.021)	(0.019)	(0.015)	(0.018)
Job search duration at survey date	0.003***	0.005**	$-$0.003	$-$0.003	0.004	$-$0.002	0.002	$-$0.000	$-$0.000
	(0.001)	(0.002)	(0.002)	(0.003)	(0.003)	(0.003)	(0.003)	(0.002)	(0.002)
Registered as unemployed	0.018**	0.003	0.003	$-$0.016	$-$0.002	$-$0.032*	$-$0.006	0.008	$-$0.026*
	(0.009)	(0.016)	(0.014)	(0.018)	(0.018)	(0.019)	(0.017)	(0.014)	(0.016)
Compulsory schooling	0.026*	0.032	$-$0.011	$-$0.002	$-$0.014	0.001	0.000	$-$0.012	$-$0.036
	(0.014)	(0.022)	(0.023)	(0.030)	(0.026)	(0.029)	(0.026)	(0.018)	(0.023)
Advanced vocational education	0.016	$-$0.126***	0.078***	$-$0.008	0.001	0.070**	0.005	0.003	0.073***
	(0.014)	(0.025)	(0.023)	(0.027)	(0.028)	(0.029)	(0.026)	(0.023)	(0.026)
University or equivalent	$-$0.004	$-$0.182***	0.149***	$-$0.002	0.003	0.048*	$-$0.012	$-$0.135***	0.144***
	(0.014)	(0.024)	(0.022)	(0.027)	(0.028)	(0.029)	(0.025)	(0.023)	(0.026)
Work experience less than 1 year	$-$0.039**	0.013	0.046*	0.063*	$-$0.074**	0.090***	$-$0.051*	0.023	$-$0.031
	(0.016)	(0.026)	(0.026)	(0.033)	(0.030)	(0.033)	(0.029)	(0.022)	(0.027)
Work experience 1–3 years	0.001	0.010	$-$0.008	0.028	$-$0.061**	0.030	$-$0.032	0.002	0.034
	(0.013)	(0.021)	(0.021)	(0.026)	(0.025)	(0.026)	(0.024)	(0.017)	(0.024)
Constant	0.458***	$-$0.807***	1.561***	0.704***	0.171*	0.733***	0.141	0.312***	0.125
	(0.049)	(0.085)	(0.086)	(0.099)	(0.100)	(0.103)	(0.094)	(0.079)	(0.095)
Observations	2,624	2,624	2,624	2,622	2,622	2,622	2,622	2,599	2,599
Adjusted R-squared	0.049	0.305	0.407	0.065	0.072	0.080	0.031	0.184	0.150

Reference categories are: basic vocational education and more than 3 years of work experience. The regressions additionally control for the level of digital skills required in the last job, function, sector, ISCO 1-digit occupation, and month of sampling. Robust standard errors in parentheses *** *p*<0.01, ** *p*<0.05, * *p*<0.1

**Role of potential skill gaps.** We find that jobseekers with higher-than-predicted digital skill ratings are less likely to aspire jobs with lower digital skill requirements than their last job and more likely to pursue jobs with higher pay. Conversely, jobseekers with lower than predicted digital skills are more likely to search for jobs with lower digital skill requirements and less likely to aspire more demanding jobs. Jobseekers who cover fewer of the 14 professional skills that are most important in the top 15 occupations search in fewer of these occupations. Moreover, they are more likely to search for jobs with less than 80% overlap in required professional skills and with wages that are more than 10% lower compared to their last occupation. Thus, it seems that they adjust their aspirations downward. Lower coverage of interdisciplinary skills correlates with a higher probability of searching for jobs with lower digital skill requirements, but it is uncorrelated with other deviations from the last job. Overall, our findings are in line with jobseekers tailoring their job search to their perceived skill profile.

**Other jobseeker characteristics.** Our results further show interesting heterogeneity with respect to other jobseeker characteristics. Women search in fewer of the top 15 occupations than men, they are more likely to aspire jobs with lower digital skill requirements, and they are less likely to search very narrowly in terms of interdisciplinary skill requirements. Interestingly, older jobseekers are more likely to pursue jobs with lower pay than their last job and less likely to pursue higher paying jobs. This speaks to older jobseekers having a higher willingness to make concessions in terms of pay. A longer job search duration at the survey date, being registered as unemployed and having the lowest level of education is associated with a broader search within the top 15 occupations. Search duration is further correlated with a higher probability of downgrading in terms of digital skill requirements. This suggests that jobseekers adjust their search strategy over time. Those registered as unemployed are less likely to deviate stronger from their last job in terms of interdisciplinary skill requirements and to pursue higher paying jobs. Highly educated jobseekers are considerably more likely to aspire jobs with higher digital skill requirements and higher pay than their last job, and they deviate more in terms of interdisciplinary skill requirements. This suggests that the last job of some high-skilled workers may have been a bad match in terms of their aspirations and that they seek to improve in this respect. Finally, jobseekers with relatively little work experience search in fewer of the top 15 jobs but considerably more broadly in terms of skill requirements.

## Search intensity and job search outcomes

As the final step of the analysis, we investigate to which extent potential skill gaps and differences in job search strategies correlate with variation in search intensity and job search outcomes. To this end, we run a set of regressions, where we sequentially study different elements of the job search process.

**Dependent variables.** In the survey, we collect information on the number of hours per week the jobseekers typically spends on job search activities, the number of applications sent out in the past four weeks, and the number of interview invitations received in the same time period. In the administrative data, we observe whether or not the jobseeker was registered with the PES six and twelve months after sampling. In Table 10, we report sample averages of these variables for the full sample and by gender. On average, jobseekers typically spent 16.5 h per week on job search activities. In the four weeks before taking the survey, they sent out 10.6 applications and received 1.5 invitations to job interviews on average. Men invest significantly more time in job search on average than women, sent out more applications and receive more interview invitations. 26% and 22% of jobseekers were registered with the PES six and twelve months after sampling, respectively. Despite different search intensity, registration status does not differ by gender.

**Table 10:** Search intensity and job search outcomes

	(1)	(2)	(3)	(4)	(5)
	All	Men	Women	Diff. (3)-(2)	P-value
*Search intensity:*					
Time for job search	16.496	17.742	15.152	$-$2.590	0.000
Number of applications	10.596	10.979	10.178	$-$0.801	0.002
Number of Job interviews	1.460	1.574	1.336	$-$0.239	0.004
*Search outcomes:*					
Registered after 6 months	0.259	0.264	0.253	$-$0.011	0.528
Registered after 12 months	0.220	0.225	0.215	$-$0.010	0.545
Observations	2,736	1,416	1,320		

The table shows sample averages of the respective item. Column (4) shows the difference in means between women and men. Column (5) reports the p-value for the test of statistical significance of this difference. Except for the first three items, all means are shares

**Explanatory variables.** The key variables of interest are again the different measures of potential skill gaps. Moreover, we are interested in the question how different search strategies are related to search intensity and outcomes. The regressions further control for the full set of available explanatory variables to account for as many potential determinants of search intensity and job search outcomes as possible. Table 11 presents the estimated coefficients for the potential skill gaps and search strategies as well as other explanatory variables where we find statistically significant associations with job search outcomes.

**Table 11:** Heterogeneity in search intensity and job search outcomes

	(1)	(2)	(3)	(4)	(5)
	Time	Number	Number	Registered	Registered
	for job	of appli-	of job	after	after
	search	cations	interviews	6 months	12 months
	Potential skill gaps				
Higher-than-predicted digital skills	0.134	$-$0.155	0.160*	$-$0.041**	0.008
	(0.596)	(0.322)	(0.095)	(0.020)	(0.021)
Lower than predicted digital skills	$-$0.351	$-$0.870***	0.203**	$-$0.013	$-$0.002
	(0.565)	(0.323)	(0.102)	(0.021)	(0.021)
Uncovered professional skills	$-$0.098	0.037	$-$0.060***	$-$0.001	$-$0.002
	(0.077)	(0.045)	(0.014)	(0.003)	(0.003)
Uncovered interdisciplinary skills	$-$0.427***	$-$0.239***	0.038	0.001	0.009**
	(0.098)	(0.059)	(0.023)	(0.003)	(0.004)
	Search strategies				
Share of top 15 searched	8.577***	2.491***	0.728***	$-$0.013	$-$0.023
	(1.209)	(0.612)	(0.194)	(0.037)	(0.037)
More than 5% lower digital skills	0.610	0.850**	0.227*	0.021	$-$0.015
	(0.592)	(0.342)	(0.119)	(0.022)	(0.021)
More than 5% higher digital skills	1.681**	0.976**	0.084	0.018	$-$0.010
	(0.694)	(0.384)	(0.115)	(0.024)	(0.025)
Overlap in professional skills <80%	1.649**	$-$0.487	$-$0.059	0.011	$-$0.012
	(0.715)	(0.376)	(0.120)	(0.024)	(0.025)
Overlap in professional skills >90%	1.020	0.533	0.119	0.038	$-$0.005
	(0.718)	(0.382)	(0.118)	(0.024)	(0.023)
Overlap in interdisciplinary skills <80%	$-$1.143*	0.067	$-$0.020	0.040*	0.009
	(0.687)	(0.373)	(0.120)	(0.024)	(0.024)
Overlap in interdisciplinary skills >90%	$-$1.676**	$-$0.814**	0.087	$-$0.041*	$-$0.035
	(0.699)	(0.385)	(0.120)	(0.024)	(0.024)
Wage more than 10% lower	0.093	0.173	0.022	$-$0.014	$-$0.024
	(0.722)	(0.416)	(0.140)	(0.025)	(0.025)
Wage more than 10% higher	$-$0.019	0.277	0.127	0.021	$-$0.005
	(0.666)	(0.344)	(0.117)	(0.022)	(0.022)
	Other characteristics				
Age	0.085***	$-$0.029**	$-$0.024***	0.004***	0.004***
	(0.024)	(0.014)	(0.005)	(0.001)	(0.001)
Job search duration at survey date	$-$0.137*	$-$0.073*	$-$0.067***	$-$0.005*	0.003
	(0.074)	(0.037)	(0.010)	(0.003)	(0.002)
Digital skills required in last job	1.916**	1.451***	0.239	$-$0.015	$-$0.058*
	(0.794)	(0.464)	(0.168)	(0.030)	(0.030)
Constant	3.504	7.320***	1.954***	0.193*	0.207**
	(2.770)	(1.598)	(0.577)	(0.103)	(0.101)
Observations	2,563	2,574	2,570	2,589	2,589
Adjusted R-squared	0.112	0.075	0.054	0.135	0.028

The regressions additionally control for gender, education, work experience, not being Swiss, being registered as unemployed, region, marital status, task content, ISCO 1-digit occupation, function and sector of last job, month of sampling. Job search duration at survey date refers to the number of days registered with the PES as jobseeker at the time of participation in the survey. Robust standard errors in parentheses *** *p*<0.01, ** *p*<0.05, * *p*<0.1

**Role of potential skill gaps.** Given that we control for search strategies, the coefficients on the skill gap measures indicate differences in search intensity and outcomes holding search strategies constant. We find that jobseekers with higher-than-predicted digital skills receive more interview invitations and deregister from the PES faster. This suggests that these jobseekers may indeed have better skills. Interestingly, jobseekers with lower than predicted digital skills also receive more interview invitations despite sending out fewer applications. However, here we do not see lower registration rates 6 and 12 months after sampling, suggesting that these jobseekers are less successful in turning interviews into job offers. There are several possible explanations. First, they may indeed have skill deficits. Second, if they are under-confident with respect to their digital skills, they might also be under-confident in general, which might show in the interview. Third, the estimated coefficients are conditional on the search strategy. If potential skill gaps affect registration rates only via different search strategies, this would render the coefficient insignificant. Lower coverage of professional skills is correlated with fewer interview invitations, but there is no significant association with being registered. In contrast, lower coverage of interdisciplinary skills is associated with lower search intensity and a higher probability of being registered with the PES 12 months after sampling, suggesting that these jobseekers may have less favourable employment prospects.

**Role of job search strategies.** A broader search in terms of number of top 15 occupations, digital skill requirements and required professional skills is associated with more time investments in search. However, only a higher share of top 15 occupations searched and downward adjustments of digital skill requirements are associated with more interviews. In contrast, both a relatively broad and a relatively narrow search in terms of interdisciplinary skill requirements are associated with lower search intensity. None of the search strategies is significantly associated with the probability of being registered with the PES 12 months after sampling, though. However, with the exception of a broader search in terms of interdisciplinary skill requirements, all point estimates are negative and reach up to $-$3.5 percentage points, pointing to lower registration rates in the longer run. For interdisciplinary skill requirements, we find that a broad search is associated with a 4-percentage-point higher probability of being registered after 6 months, suggesting a longer search duration, while the probability is 4 percentage points lower with a narrow search, indicating that these jobseekers deregister faster. Thus, searching quite narrowly in terms of overlap in interdisciplinary skills seems to be associated with more favourable job search outcomes, while the reverse seems to be the case for a broader search.

**Other characteristics.** Conditional on search strategies and potential skill gaps, we find that older workers invest more time in job search but send out fewer applications. They receive fewer interview invitations and are more likely to be registered with the PES 6 and 12 months after sampling. This suggests that older workers have more difficulty finding a job, holding everything else constant. A longer search duration at the survey date is associated with lower search intensity and fewer interview invitations, which is in line with this group being negatively selected. However, they are more likely to deregister after six months, which may be mechanical given their already longer search duration. Jobseekers whose last job required more digital skills search more intensely, and they are less likely to be registered with the PES 12 months after sampling, suggesting that jobseekers from more digital-skill-intensive jobs may have better employment prospects.

**Robustness.** Table 11 looks at different elements of the job search process, and they vary with the characteristics of the jobseekers and their search strategies. To isolate the additional explanatory power of these variables conditional on the previous step of the job search process, we run a second set of regressions where we control for the dependent variables of all previous steps as potential mediators. For example, we first study search intensity and then search outcomes conditional on search intensity. Table 18 in Appendix F presents the results. In line with expectations, we find that a longer time input to search is associated with more applications and these, in turn, with more interview invitations, which are associated with lower registration rates 6 and 12 months after sampling. However, the estimated coefficients for our variables of interest remain very similar, suggesting that they capture direct associations with the respective dependent variable.

## Conclusion

This paper uses novel linked survey and administrative data for jobseekers in Switzerland to study jobseekers skills and job search behaviour. We document how jobseekers perceive their digital, professional and interdisciplinary skills, and use these self-assessments to construct a measure of potential skill gaps. We then show how occupational differences in wages, labour market tightness, and skill requirements relative to the last job relate to the probability that a jobseeker searches in a given occupations. In the final step, we investigate how potential skill gaps, job search strategies and job search outcomes are related.

We document considerable heterogeneity in skill self-assessments after accounting for occupational sorting and educational attainment. In line with previous evidence on a gender divide in digital skills and skill investments (see Van Laar et al., 2017, for a review), we find that women rate their overall and advanced digital skills significantly lower than men. They also report lower ratings for professional skills but higher ratings for interdisciplinary skills. This is in line with evidence that women tend to under-estimate their IQ, whereas men tend to over-estimate it, while the reverse is observed for emotional intelligence (Reilly et al., 2022; Furnham & Robinson, 2023). We further document a negative association of skill ratings with age for all skill dimensions except interdisciplinary skills, which is particularly large for digital skills. This is in line with previous findings (e.g. Bhattacharjee et al., 2020) and corroborates concerns that older jobseekers may be less well prepared for the digital transformation. Our measures of potential skill gaps confirm that women and older jobseekers are most at risk of lacking digital skills, while low education and little work experience are risk factors associated with lacking professional and interdisciplinary skills.

Our analysis of job search strategies reveals that jobseekers prefer jobs that are more similar to their last job. They are particularly reluctant to accept wage losses, especially men, while older jobseekers seem to have a higher willingness to make concessions in terms of pay. Jobseekers are less likely to pursue jobs the more their skill profiles differ from their last job, even among jobs with relatively large skill overlap. However, we find that jobseekers with potential skill gaps are more likely to adjust their search strategy to different skill profiles. Conditional on search strategies, we document that jobseekers with higher-than-predicted digital skills deregister from the PES faster, while potential digital skill deficits and lower coverage of professional skills are uncorrelated with registration rates. In contrast, lower coverage of interdisciplinary skills is associated with a higher probability of being registered with the PES 12 months after sampling.

Our results identify risk groups that potentially lack important skills and provide novel insights on how potential skill gaps, job search strategies and job search outcomes are related. These insights are important inputs for policy makers and practitioners in the PES, who seek to support jobseekers in the digital transformation and labour markets that are characterized by rapidly changing skill requirements. Our results inform for which jobseekers investments in digital and other skills might be necessary, and they identify scope for improvement in job search strategies. They also show the value of collecting data on skill self-assessments and measuring skill mismatch on the individual level. Future research should shed light on jobseekers’ actual skills, which allows measuring biases in beliefs and identifying skill deficits more accurately.

## Data Availability

The survey data will made publicly available in anonymized form after completion of the NRP77 project. After publication of the survey data, researchers can apply for linking the survey data with the administrative data at the Federal Statistical Office according to the procedure described here: https://www.bfs.admin.ch/bfs/de/home/dienstleistungen/datenverknuepfungen/fuer-dritte.html.

## Survey question on digital skills

See Fig. 4.

## Correlation between Survey Items

See Table 12.

**Table 12:** Correlations between Digital Skill Items

	(1)	(2)	(3)	(4)	(5)	(6)	(7)	(8)	(9)	(10)	(11)
(1) Using digital devices	1.000										
(2) Handling information, data and digital content	0.845	1.000									
(3) Communicating, collaborating and sharing information	0.790	0.823	1.000								
(4) Making online purchases or sales	0.633	0.620	0.656	1.000							
(5) Developing digital content and programming	0.400	0.435	0.426	0.390	1.000						
(6) Automating processes	0.465	0.517	0.507	0.451	0.630	1.000					
(7) Protecting devices and personal data	0.457	0.482	0.501	0.429	0.503	0.588	1.000				
(8) Solving technical problems	0.506	0.514	0.515	0.390	0.590	0.582	0.621	1.000			
(9) Solving tasks or problems using digital technologies	0.617	0.645	0.656	0.506	0.569	0.624	0.621	0.757	1.000		
(10) Recognizing digital skill deficits	0.553	0.588	0.597	0.461	0.456	0.514	0.537	0.573	0.673	1.000	
(11) Digital competencies overall	0.707	0.727	0.725	0.569	0.571	0.616	0.600	0.684	0.800	0.757	1.000

The table shows the pairwise correlations between the different digital skill items

## Distribution of self-assessed digital skills

See Fig. 5.

## Digital skill requirements of last job

See Fig. 6.

## Descriptive statistics for top 15 occupations

See Tables 13 and 14.

**Table 13:** Descriptive statistics for top 15 occupations for men

	(1)	(2)	(3)	(4)	(5)	(6)	(7)
	Mean	Std.	.25	.50	.75	Min	Max
Difference in digital skills	0.006	0.260	$-$0.143	0.000	0.087	$-$0.667	1.833
Difference in basic digital skills	$-$0.002	0.234	$-$0.148	0.000	0.095	$-$0.661	1.949
Difference in advanced digital skills	$-$0.003	0.247	$-$0.151	0.000	0.092	$-$1.000	1.949
Overlap in professional skills	0.813	0.206	0.705	0.885	0.974	0.000	1.000
Overlap in interdisciplinary skills	0.775	0.228	0.667	0.833	1.000	0.000	1.000
Wage difference	$-$0.012	0.262	$-$0.161	0.000	0.093	$-$0.717	1.835
Vacancy difference	13.850	97.689	$-$0.796	0.000	2.343	$-$1.000	3292
Jobseeker difference	5.282	37.186	$-$0.714	0.000	1.884	$-$1.000	3047
Observations	21,345						
Individuals	1,423						

The sample consists of all occupations in the list of 15 provided to jobseekers. All differences are measured in percent/100 of the level of the last job. Overlap measures the share that the last job has in common with the respective occupation

**Table 14:** Descriptive statistics for top 15 occupations for women

	(1)	(2)	(3)	(4)	(5)	(6)	(7)
	Mean	Std.	.25	.50	.75	Min	Max
Difference in digital skills	0.018	0.245	$-$0.130	0.000	0.150	$-$0.643	2.000
Difference in basic digital skills	0.019	0.233	$-$0.118	0.000	0.134	$-$0.620	1.515
Difference in advanced digital skills	0.009	0.224	$-$0.114	0.000	0.106	$-$1.000	1.833
Overlap in professional skills	0.819	0.213	0.744	0.897	0.974	0.000	1.000
Overlap in interdisciplinary skills	0.794	0.209	0.708	0.833	1.000	0.000	1.000
Wage difference	0.056	0.276	$-$0.091	0.000	0.167	$-$0.717	2.144
Vacancy difference	11.662	99.330	$-$0.853	$-$0.298	1.347	$-$1.000	3292
Jobseeker difference	4.346	36.672	$-$0.879	$-$0.051	0.798	$-$1.000	2301
Observations	19,860						
Individuals	1,324						

The sample consists of all occupations in the list of 15 provided to jobseekers. All differences are measured in percent/100 of the level of the last job. Overlap measures the share that the last job has in common with the respective occupation

## Additional estimation results

See Tables 15, 16, 17 and 18.

**Table 15:** Heterogeneity in self-assessed skills: robustness

	(1)	(2)	(3)	(4)	(5)	(6)
	Intention to strengthen .					
	Overall	Profes-	Interdis-	Overall	Profes-	Interdis-
	digital	sional	ciplinary	digital	sional	ciplinary
	skills	skills	skills	skills	skills	skills
Higher-than-predicted digital skills	$-$0.006	0.060**	0.079***	$-$0.016	0.033	0.073***
	(0.025)	(0.024)	(0.025)	(0.025)	(0.024)	(0.025)
Lower than predicted digital skills	$-$0.013	$-$0.015	$-$0.009	$-$0.019	$-$0.025	$-$0.003
	(0.026)	(0.026)	(0.027)	(0.026)	(0.025)	(0.027)
Lacking professional skills	$-$0.009**	$-$0.002	$-$0.009**	$-$0.008**	$-$0.001	$-$0.009**
	(0.004)	(0.003)	(0.004)	(0.004)	(0.003)	(0.004)
Lacking interdisciplinary skills	$-$0.005	$-$0.005	$-$0.015***	$-$0.007	$-$0.009**	$-$0.015***
	(0.005)	(0.004)	(0.005)	(0.005)	(0.005)	(0.005)
Other covariates	No	No	No	Yes	Yes	Yes
Observations	2,314	2,317	2,261	2,314	2,317	2,261
Adjusted R-squared	0.005	0.006	0.025	0.016	0.040	0.037

Control variables include age, indicator for not being Swiss, search duration at survey data, being registered as unemployed, education, work experience, function and sector of last job, month of sampling. Current job search duration refers to the number of days registered with the PES as jobseeker at the time of participation in the survey. Robust standard errors in parentheses **** p*<0.01, ** * p*<0.05, * * p*<0.1

**Table 16:** Heterogeneity in self-assessed skills: Robustness

	(1)	(2)	(3)	(4)	(5)
	Overall	Basic	Advanced	Profes-	Interdis-
	digital	digital	digital	sional	ciplinary
	skills	skills	skills	skills	skills
Digital skills required in last job	0.509***	0.406***	0.435***	$-$0.004	0.077***
	(0.072)	(0.055)	(0.073)	(0.026)	(0.016)
Management function	0.100	0.147***	0.079	0.159***	0.161***
	(0.074)	(0.053)	(0.076)	(0.027)	(0.016)
Support function	$-$0.206**	$-$0.110*	$-$0.120	$-$0.072***	$-$0.061***
	(0.081)	(0.066)	(0.075)	(0.025)	(0.017)
Low-tech manufacturing	$-$0.001	0.106	0.029	$-$0.034	$-$0.018
	(0.099)	(0.073)	(0.093)	(0.032)	(0.021)
High-tech manufacturing	0.329***	0.247***	0.230**	0.034	0.052**
	(0.092)	(0.069)	(0.101)	(0.035)	(0.022)
Knowledge-intensive services	$-$0.029	$-$0.015	$-$0.029	$-$0.010	0.033***
	(0.060)	(0.047)	(0.057)	(0.019)	(0.013)
Construction	$-$0.433***	$-$0.471***	$-$0.387***	$-$0.126***	$-$0.113***
	(0.131)	(0.110)	(0.125)	(0.040)	(0.029)
Compulsory schooling	$-$0.196**	$-$0.234***	$-$0.124	$-$0.055**	$-$0.042**
	(0.087)	(0.072)	(0.082)	(0.027)	(0.018)
Advanced vocational education	0.181***	0.206***	0.062	0.102***	0.025
	(0.070)	(0.055)	(0.070)	(0.024)	(0.016)
University or equivalent	0.264***	0.233***	0.084	0.104***	0.074***
	(0.070)	(0.051)	(0.070)	(0.024)	(0.016)
Work experience less than 1 year	$-$0.010	0.042	0.036	$-$0.142***	$-$0.042**
	(0.096)	(0.075)	(0.089)	(0.030)	(0.020)
Work experience 1–3 years	$-$0.013	0.006	0.035	$-$0.077***	$-$0.048***
	(0.075)	(0.059)	(0.071)	(0.024)	(0.016)
Job search duration at survey date	0.001	$-$0.003	0.007	$-$0.002	$-$0.006***
	(0.007)	(0.006)	(0.007)	(0.002)	(0.002)
Registered as unemployed	0.057	0.017	0.032	$-$0.021	$-$0.057***
	(0.049)	(0.039)	(0.048)	(0.016)	(0.011)
Female	$-$0.222***	$-$0.078*	$-$0.286***	$-$0.116***	0.049***
	(0.053)	(0.043)	(0.052)	(0.018)	(0.011)
Age	$-$0.018***	$-$0.022***	$-$0.019***	$-$0.004***	0.000
	(0.003)	(0.002)	(0.003)	(0.001)	(0.001)
Not Swiss	0.130**	0.048	0.204***	0.167***	0.072***
	(0.054)	(0.042)	(0.053)	(0.018)	(0.012)
Constant	4.635***	5.983***	4.463***	5.653***	6.053***
	(0.260)	(0.210)	(0.257)	(0.087)	(0.057)
Observations	2,532	2,617	2,587	34,463	36,168
Adjusted R-squared	0.128	0.206	0.075	0.019	0.028

Robust standard errors in parentheses. *** * p*<0.01, ** * p*<0.05, * * p*<0.1. For professional and interdisciplinary skills, there are 14 skill items per person and standard errors are clustered at the individual level. The regressions additionally control for ISCO 1-digit occupations, an indicator for being married, region and month of sampling

**Table 17:** Search strategies and occupation characteristics

	Share of	Digital skills		Professional skills		Interdisciplinary skills		Wage	
	top 15	More than	More than	Overlap	Overlap	Overlap	Overlap	More than	More than
	searched	5% lower	5% higher	<80%	>90%	<80%	>90%	10% lower	10% higher
	(1)	(2)	(3)	(4)	(5)	(6)	(7)	(8)	(9)
Wage difference	$-$0.0418	$-$0.4268***	0.5742***	0.0251	$-$0.0225	$-$0.1577***	$-$0.1607***		
	(0.0284)	(0.0527)	(0.0496)	(0.0473)	(0.0472)	(0.0502)	(0.0408)		
Vacancy difference	$-$0.0001*	0.0001	0.0001	$-$0.0000	0.0002*	$-$0.0002	0.0002*	0.0001*	$-$0.0000
	(0.0000)	(0.0001)	(0.0001)	(0.0001)	(0.0001)	(0.0001)	(0.0001)	(0.0001)	(0.0001)
Jobseeker difference	$-$0.0009***	0.0021***	$-$0.0027***	0.0038***	$-$0.0025***	0.0014*	$-$0.0004	0.0022***	0.0004
	(0.0003)	(0.0006)	(0.0007)	(0.0007)	(0.0007)	(0.0008)	(0.0006)	(0.0006)	(0.0006)
Difference in digital skills	$-$0.0671**			0.2964***	$-$0.0813*	0.0321	0.2516***	$-$0.4157***	0.3705***
	(0.0331)			(0.0493)	(0.0441)	(0.0464)	(0.0390)	(0.0426)	(0.0549)
Gap in professional skills	0.1321**	$-$0.4762***	1.4680***			2.6710***	$-$2.1892***	$-$0.5705***	0.0500
	(0.0603)	(0.1243)	(0.1143)			(0.0715)	(0.0819)	(0.1131)	(0.1071)
Gap in interdisciplinary skills	0.1061**	0.5209***	$-$0.4301***	1.9970***	$-$1.7984***			0.9846***	0.0796
	(0.0534)	(0.1088)	(0.0872)	(0.0746)	(0.0891)			(0.1049)	(0.0843)
Constant	0.4316***	0.3192***	0.1676***	$-$0.0851***	0.6694***	$-$0.0754***	0.6047***	0.0785***	0.2049***
	(0.0090)	(0.0163)	(0.0154)	(0.0109)	(0.0173)	(0.0117)	(0.0164)	(0.0122)	(0.0149)
Observations	2.574	2.574	2.574	2.574	2.574	2.574	2.574	2.574	2.574
Adjusted R-squared	0.0138	0.0372	0.1157	0.3495	0.2415	0.3935	0.2933	0.1006	0.0231

Robust standard errors in parentheses *** *p*<0.01, ** *p*<0.05, * *p*<0.1

**Table 18:** Determinants of search intensity and job search outcomes

	(1)	(2)	(3)	(4)	(5)
	Time	Number	Number	Registered	Registered
	for job	of appli-	of job	after	after
	search	cations	interviews	6 months	12 months
Higher-than-predicted digital skills	0.134	$-$0.188	0.162*	$-$0.037*	0.012
	(0.596)	(0.310)	(0.092)	(0.020)	(0.021)
Lower than predicted digital skills	$-$0.351	$-$0.805**	0.265***	$-$0.004	0.003
	(0.565)	(0.314)	(0.102)	(0.021)	(0.021)
Uncovered professional skills	$-$0.098	0.041	$-$0.059***	$-$0.002	$-$0.002
	(0.077)	(0.044)	(0.014)	(0.003)	(0.003)
Uncovered interdisciplinary skills	$-$0.427***	$-$0.177***	0.053**	0.002	0.009**
	(0.098)	(0.059)	(0.023)	(0.003)	(0.004)
Share of top 15 searched	8.577***	1.376**	0.546***	0.004	$-$0.009
	(1.209)	(0.595)	(0.197)	(0.038)	(0.038)
More than 5% lower digital skills	0.610	0.832**	0.176	0.025	$-$0.009
	(0.592)	(0.330)	(0.115)	(0.022)	(0.022)
More than 5% higher digital skills	1.681**	0.768**	0.013	0.014	$-$0.011
	(0.694)	(0.374)	(0.113)	(0.025)	(0.025)
Overlap in professional skills <80%	1.649**	$-$0.809**	$-$0.054	0.017	$-$0.013
	(0.715)	(0.368)	(0.119)	(0.024)	(0.026)
Overlap in professional skills >90%	1.020	0.433	0.040	0.041*	$-$0.002
	(0.718)	(0.373)	(0.114)	(0.024)	(0.024)
Overlap in interdisciplinary skills <80%	$-$1.143*	0.288	$-$0.003	0.039	0.009
	(0.687)	(0.365)	(0.117)	(0.024)	(0.024)
Overlap in interdisciplinary skills >90%	$-$1.676**	$-$0.601	0.183	$-$0.040	$-$0.039
	(0.699)	(0.376)	(0.118)	(0.024)	(0.025)
Wage more than 10% lower	0.093	0.091	$-$0.001	$-$0.016	$-$0.027
	(0.722)	(0.400)	(0.135)	(0.026)	(0.025)
Wage more than 10% higher	$-$0.019	0.229	0.140	0.018	$-$0.011
	(0.666)	(0.330)	(0.114)	(0.022)	(0.022)
Female	$-$1.480***	$-$0.711**	$-$0.187**	$-$0.014	0.010
	(0.530)	(0.276)	(0.093)	(0.018)	(0.018)
Age	0.085***	$-$0.041***	$-$0.023***	0.004***	0.004***
	(0.024)	(0.013)	(0.005)	(0.001)	(0.001)
Not Swiss	1.906***	0.102	0.001	$-$0.002	0.001
	(0.526)	(0.284)	(0.095)	(0.019)	(0.019)
Job search duration at survey date	$-$0.137*	$-$0.053	$-$0.063***	$-$0.005*	0.003
	(0.074)	(0.036)	(0.010)	(0.003)	(0.003)
Registered as unemployed	1.691***	0.381	0.045	0.024	$-$0.007
	(0.463)	(0.254)	(0.087)	(0.017)	(0.017)
Digital skills required in last job	1.916**	1.129**	0.135	$-$0.017	$-$0.054*
	(0.794)	(0.452)	(0.166)	(0.031)	(0.030)
Time for job search		0.139***	0.006	$-$0.000	0.000
		(0.012)	(0.004)	(0.001)	(0.001)
Number of applications			0.061***	0.001	$-$0.001
			(0.008)	(0.001)	(0.001)
Number of job interviews				$-$0.012***	$-$0.013***
				(0.004)	(0.004)
Constant	3.504	6.831***	1.467***	0.198*	0.217**
	(2.770)	(1.565)	(0.565)	(0.103)	(0.102)
Observations	2,563	2,548	2,536	2,527	2,527
Adjusted R-squared	0.112	0.137	0.093	0.138	0.031

The regressions additionally control for region, marital status, task content, ISCO 1-digit occupation, function and sector of last job, month of sampling, indicator for not being registered at the online job search platform of the PES. Current job search duration refers to the number of days registered with the PES as jobseeker at the time of participation in the survey. Robust standard errors in parentheses *** *p*<0.01, ** *p*<0.05, * *p*<0.1

## Footnotes

1 Kuhn and Skuterud (2004); Krueger et al. (2011); Kuhn and Mansour (2014); Cottier et al. (2016); Mukoyama et al. (2018); Arni and Schiprowski (2019); Gee (2019); Faberman and Kudlyak (2019); DellaVigna et al. (2021); Kircher (2022); Zuchuat (2023).

2 Kudlyak et al. (2013); Sahin et al. (2014); Guglielminetti et al. (2015); Altmann et al. (2018); Belot et al. (2018); Marinescu and Rathelot (2018); Cottier et al. (2020); Belot et al. (2022); Dhia et al. (2022); van der Klaauw and Vethaak (2022); Mueller-Gastell and Pedulla (2023).

3 The excluded REOs comprise those that participated in other studies or surveys in 2022 and 2023.

4 Occupations are based on the Swiss classification of occupations (SBN) using 6-digit codes. We also observe the corresponding International Standard Classification of Occupations (ISCO-08) code on the 4-digit level. Both allow us to merge occupation-level data on skill requirements, wages, vacancies and number of jobseekers.

5 Analysing such text data and mapping them into the ISCO classification is complex and time-consuming, and we have not done so, yet.

6 https://bis.ams.or.at/bis/.

7 We obtained the information how skill lists and digital skill levels are compiled in an online meeting with several representatives of the AMS that took place on 29 May 2024.

8 We abstain from showing the questions for professional and interdisciplinary skills as they were personalized for each jobseeker.

9 We compile a list of all skills required in the top 15 occupations and use the 14 skills that are most frequent within the top 15 occupations.

10 Note that the numbers of vacancies and jobseekers are correlated positively at a value of .219 (p-value .000) because they are both related to the “size” of an occupation.
